# LncRNA TSPEAR-AS2 Maintains the Stemness of Gastric Cancer Stem Cells by Regulating the miR-15a-5p/CCND1 Axis

**DOI:** 10.3390/biom15091227

**Published:** 2025-08-26

**Authors:** Qiong Li, Yanan Wang, Liyang Chen, Yan Shen, Shijiao Zhang, Dengyuan Yue, Xiaowei Chen

**Affiliations:** 1 School of Elderly Care Services and Management, Nanjing University of Chinese Medicine, Nanjing 210023, China; liqiong@njucm.edu.cn; 2 School of Nursing, Nanjing University of Chinese Medicine, Nanjing 210023, China; 3 Key Laboratory of Environmental Medicine Engineering, Ministry of Education, School of Public Health, Southeast University, Nanjing 210009, China; 4 Department of Epidemiology and Health Statistics, School of Public Health, Southeast University, Nanjing 210009, China; 5 School of Aging Industry, Nanjing University of Chinese Medicine, Nanjing 210023, China

**Keywords:** gastric cancer, gastric cancer stem cells, LncRNA, ceRNA, stemness

## Abstract

Cancer stem cells (CSCs), a subpopulation of tumor cells endowed with self-renewal capacity, drive cancer initiation and progression. While long non-coding RNAs (lncRNAs) are increasingly recognized as critical regulators of CSC stemness, their specific roles in gastric cancer stem cells (GCSCs) remain poorly understood. This study investigates the functional significance of lncRNA TSPEAR-AS2 in modulating GCSC properties and uncovers its underlying molecular mechanisms. Through integrated whole-transcriptome sequencing, bioinformatics analysis, and validation in 48 paired gastric cancer tissues and adjacent normal tissues, TSPEAR-AS2 was identified as a differentially expressed lncRNA upregulated in both GCSCs and tumor samples. Functional experiments revealed that TSPEAR-AS2 overexpression significantly enhanced GCSC sphere-forming ability, proliferation, cell cycle progression, epithelial–mesenchymal transition (EMT), and expression of stemness markers (CD54, CD44, OCT4, NANOG, and SOX2) while suppressing apoptosis. Conversely, TSPEAR-AS2 knockdown attenuated these malignant phenotypes. In vivo tumorigenicity assays in nude mice further confirmed that TSPEAR-AS2 promotes tumor growth, with overexpression accelerating and knockdown inhibiting tumor formation. Mechanistically, bioinformatics predictions and dual-luciferase reporter assays established TSPEAR-AS2 as a competing endogenous RNA (ceRNA) that sponges miR-15a-5p, thereby derepressing the miR-15a-5p target gene CCND1. Rescue experiments demonstrated that overexpression of miR-15a-5p phenocopied TSPEAR-AS2 knockdown, reducing GCSC stemness, while miR-15a-5p inhibition rescued the effects of TSPEAR-AS2 suppression. Collectively, these findings reveal a novel TSPEAR-AS2/miR-15a-5p/CCND1 regulatory axis that sustains GCSC stemness and tumorigenicity. These results highlight TSPEAR-AS2 as a potential therapeutic target for eradicating gastric cancer stem cells and improving clinical outcomes.

## 1. Introduction

Gastric cancer (GC) remains a significant threat to human health and impedes socioeconomic development [1]. According to the latest statistics from the International Agency for Research on Cancer (IARC), in 2020, the global incidence of GC reached 1,089,000 new cases, making it the fifth most common cancer and the fourth leading cause of cancer-related death worldwide, posing a significant threat to public health [2]. Multiple factors influence the development of GC, including Helicobacter pylori infection [3], age [4], sex [5], dietary habits [6], and family history [7], complicating its prevention and treatment. In the early stages, GC presents insidiously and is challenging to diagnose [8]. With the rapid development of medical technology, the five-year survival rate of GC patients has exceeded 30% through means such as imaging techniques, neoadjuvant therapy, and molecular biopsy staging at the initial diagnosis [9]. However, the prognosis of most patients remains poor. Therefore, there is an urgent need to develop reliable prevention and treatment strategies for GC.

Cancer stem cells (CSCs) are a subgroup of tumor cells [10], initially identified in hematologic malignancies [11] and subsequently discovered in breast cancer [12], adenocarcinoma [13], ovarian cancer [14], liver cancer [15], lung cancer [16], gastric cancer [17], and other solid malignant tumors [18]. CSCs are capable of self-renewal, are highly tumorigenic, are highly metastatic, possess multi-directional differentiation, and exhibit multi-drug resistance [19,20]. The key features of gastric cancer stem cells (GCSCs) are unlimited proliferative potential, self-renewal, and resistance to apoptosis, which are considered the primary drivers of drug resistance, metastasis, and recurrence in gastric cancer [21,22].

Long non-coding RNAs (LncRNAs) are RNA molecules with transcripts longer than 200 nucleotides [23,24]. As non-coding RNA molecules, LncRNAs do not encode proteins directly but play an indispensable role in the biological processes of the organism [25]. LncRNAs can mediate epigenetic, transcriptional, and post-transcriptional regulation [26], thus contributing to the regulation of cellular homeostasis and gene expression and playing a central role in cellular processes, biological development, and disease progression [27]. Several recent studies have shown that LncRNAs are crucial in cancer because they regulate CSCs, serving as potential diagnostic and prognostic biomarkers and therapeutic targets for malignant tumors [28,29].

MicroRNAs (miRNAs) are a class of endogenous, small RNAs approximately 22 nucleotides in length that play a variety of important regulatory roles within the cell [30,31]. As competitive endogenous RNAs (ceRNAs), LncRNAs bind to miRNAs and act as miRNA sponges, thereby modulating miRNA expression and indirectly affecting the expression of miRNA-associated target genes, leading to regulatory effects on cancer [29,30,31,32,33].

In this study, we screened LncRNAs closely associated with GCSCs based on whole-transcriptome sequencing using bioinformatics methods and validated them in GC tissues and GCSCs. We then investigated the effect of these LncRNAs on the stemness-related functions of GCSCs using a range of molecular biology techniques. We further explored the downstream miRNA targets of LncRNAs to elucidate their mechanism of action. These data may provide targets for the prevention and effective treatment of GC, offer new insights and directions for advancing etiological research, and provide a theoretical basis for the potential clinical application of relevant GCSCs.

## 2. Materials and Methods

### 2.1. Patients and Tissue Samples

Tumor tissue and adjacent normal tissue samples from 48 pairs of GC patients were obtained from Zhongda Hospital, Southeast University, with approval from the Ethics Committee. All subjects signed informed consent forms. The tissues were collected following surgical excision from individuals who had not undergone prior radiotherapy or chemotherapy. The samples were stored at −80 °C.

**Figure 1:**
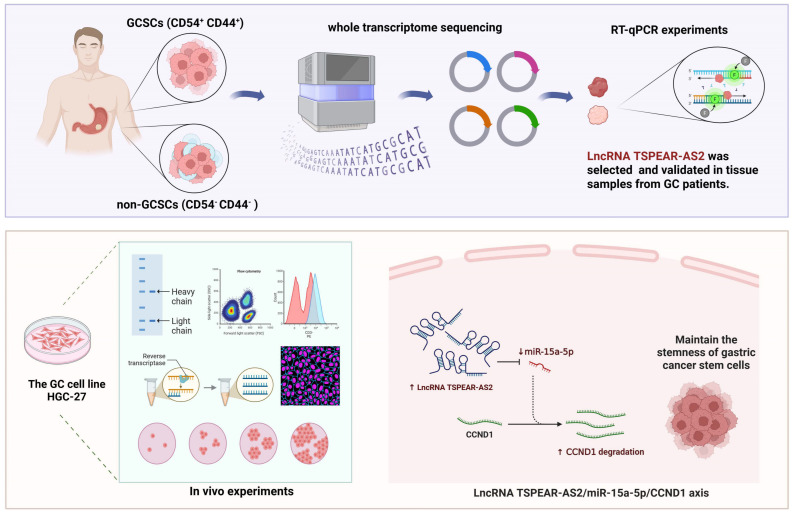
The flowchart of this study.

Inclusion criteria were 1. patients diagnosed with GC; 2. patients who had not received radiotherapy or chemotherapy before surgery; and 3. patients in good overall health and eligible for surgery. Exclusion criteria were 1. patients with concurrent tumors or other diseases of the digestive system; 2. patients deemed unsuitable for radical GC surgery prior to the operation; and 3. patients who died in the perioperative period due to surgical complications. This study’s flowchart is presented in Figure 1.

### 2.2. Cell Culture

The GC cell line HGC-27 (RRID: CVCL 1279) was purchased from Saiku Biological Company (Guangzhou, China) and authenticated using STR profiling. Cell resuscitation and culture were performed by adding 8 mL of RPMI-1640 medium containing 1% penicillin–streptomycin solution and 10% fetal bovine serum to a sterile Petri dish. The thawed cells were then added to the Petri dish and placed in a cell incubator with 5% carbon dioxide and saturated humidity at 37 °C for culturing. We changed the culture medium daily. Based on the cell growth situation, we passaged the cells every 2–3 days. Cell passage involved the old culture medium being discarded from the culture dish, and the cells were washed three times with PBS solution. We added 1 mL of trypsin digestion solution, observed under an inverted microscope after digestion at room temperature for about 2 min. When the cells became round, shrank, and started to detach, we immediately added an appropriate amount of RPMI-1640 complete medium to the culture dish to terminate the digestion. After the cells were completely detached from the dish wall, we transferred the digestion mixture containing the cells to a 15 mL sterile centrifuge tube and centrifuged them at 800 rpm for 5 min. We discarded the supernatant, resuspended the cells in RPMI-1640 complete medium, and evenly distributed them into two new culture dishes, each containing 8 mL of RPMI-1640 complete medium. Sorted GCSCs were cultured in serum-free DMEM/F12 complete medium supplemented with growth factors, including B-27 (2%), N-2 (1%), epidermal growth factor (EGF, 20 ng/mL), and basic fibroblast growth factor (b-FGF, 20 ng/mL). All cells were cultured in an incubator with 5% CO_2_ at 37 °C.

### 2.3. Isolation of CD54^+^ CD44^+^ and CD54^−^ CD44^−^ Populations

The tumor tissues of GC patients and HGC-27 cells in the logarithmic growth phase were separately prepared into single-cell suspensions. According to the cell count and the instructions for the flow cytometry antibodies, corresponding volumes of CD44-FITC and CD54-PE antibodies (5 μL of CD44-FITC and 5 μL of CD54-PE antibodies for 1 × 10^6^ cells) were added to the cell and PBS mixture. Meanwhile, isotype controls were set up for each antibody, namely, the CD44 single antibody group (incubated with CD44-FITC antibody), the CD54 single antibody group (incubated with CD54-PE antibody), and the control group (without antibody incubation). The samples were incubated in the dark at 4 °C for 30 min (mixed via pipetting once every 10 min). After the reaction was completed, 1 mL of PBS was added to wash the cells, and the samples were centrifuged at 800 rpm for 5 min. After washing, 500 μL of PBS was added to resuspend the cell pellets, and the suspension was filtered through a single-cell filter into flow cytometry tubes to obtain single-cell suspensions. The suspensions were sorted via flow cytometry to obtain a CD54^+^ CD44^+^ double-positive cell subpopulation and a CD54^−^ CD44^−^ double-negative cell subpopulation, which were, respectively, identified as GCSCs and non-gastric cancer stem cells (non-GCSCs). GCSCs derived from tumor tissues were utilized for whole-transcriptome sequencing. The HGC-27-derived GCSCs were used for experimental studies.

### 2.4. Quantitative Real-Time Polymerase Chain Reaction (RT-qPCR) Analysis

Total RNA was extracted from GC tissues and cells using RNA-easy reagent (Vazyme, Nanjing, China). The NanoDrop 2000 spectrophotometer (Thermo Fisher Scientific, Waltham, MA, USA) was used to detect the purity and concentration of the total RNA. cDNA was synthesized using the PrimeScript™ RT reagent kit (GeneStar, Beijing, China). The qPCR reaction was prepared in a 10 μL mixture containing 1 μL of diluted cDNA, 5 μL of 2 × AceQ Universal SYBR qPCR Master Mix (Vazyme, Nanjing, China), 4 μL of DEPC water, and 0.2 μL each of forward (FP) and reverse primers (RP). The reaction was performed on a StepOnePlus PCR System (Applied Biosystems, Waltham, MA, USA) with the following cycling conditions: initial pre-denaturation at 95 °C for 5 min, followed by 40 cycles of 95 °C for 10 s and 60 °C for 30 s. Relative transcript expression was calculated using the 2^−ΔΔCt^ method. All primers were synthesized by Sangon Biotech (Shanghai, China). The primer sequences are listed in Supplementary Table S1.

### 2.5. Construction and Transfection

The TSPEAR-AS2 knockdown and overexpression lentiviruses used in this experiment were generated by Genechem (Shanghai, China). The miR-15a-5p mimic and inhibitor constructs were developed by Hanbio Biotechnology (Shanghai, China). We adjusted the density of the GCSCs to 3 × 10^5^ cells/mL. The cell suspension was added to an ultra-low attachment six-well plate at a volume of 1 mL per well. The culture plate was placed in a cell incubator with 5% CO_2_ at 37 °C for 6–12 h to ensure that the cell confluence was between 30% and 50%. We took out the lentivirus (previously thawed on ice) and, based on the MOI explored in the preliminary experiment, added an appropriate volume to the wells for infection (volume of virus per well (µL) = MOI × number of cells/virus titer (TU/mL) × 1000). After 4 h of incubation, we supplemented each well with 1 mL of DMEM/F12 complete medium and continued culturing until the medium changed the next day. The cell infection situation was observed under a microscope daily. When it reached the infection peak after about 72 h, we added puromycin and continued to culture it to screen out stably transfected cell lines. The state of the transfected cells was observed under a microscope every 12 h, and fresh medium was added. Screening was performed with puromycin until the cell growth state was stable and good. We passaged, expanded the culture, and cryopreserved the stably transfected cells.

### 2.6. Western Blotting Assay

Protein samples were extracted from GCSCs and tumor tissue using a RIPA lysis buffer (EpiZyme Biotechnology, Shanghai, China). First, we prepared gel in a glass plate according to the instructions of the gel preparation kit (EpiZyme Biotechnology, Shanghai, China). We placed the glass plate in an electrophoresis tank and added the electrophoresis buffer. The protein samples and protein marker were loaded into the loading wells. Electrophoresis was performed at a constant voltage of 100 V. The PVDF membrane was activated with methanol. The components were placed in the transfer cassette in the following order: sponge, filter paper, gel, PVDF membrane, filter paper, and sponge. The transfer was conducted at a constant voltage of 100 V under ice bath conditions. After the transfer was complete, the PVDF membrane was placed in an incubation box and washed three times with TBST. The blocking solution was added, and the incubation box was placed on a shaker for 1 h of blocking at room temperature. The primary antibody incubation solution was added and incubated overnight in a chromatography cabinet at 4 °C. The secondary antibody solution was added and incubated at room temperature for 1 h. The developer was pre-cooled by turning it on. The washed PVDF membrane was blotted dry with dust-free filter paper and then immersed in the luminescent solution. A chemiluminescence image analysis system was used to take pictures, analyze the results, and save the data.

### 2.7. Immunohistochemistry (IHC)

The tumor tissues of nude mice fixed in 4% paraformaldehyde were paraffin-embedded. The paraffin-embedded tissues were sectioned into slices approximately 4 μm thick using a microtome. The slices were placed in an oven at 60 °C for 2 h and then successively immersed in xylene, 100% ethanol, 95% ethanol, 90% ethanol, 85% ethanol, 80% ethanol, and distilled water for dewaxing. The slices were immersed in 3% hydrogen peroxide for about 10 min. After washing, the slices were steamed in water containing citrate buffer until boiling, cooled, and steamed again. After cooling, the slices were washed with PBS, blotted dry, and immediately blocked with an appropriate amount of serum at 37 °C. After blocking, the primary antibody was added and incubated overnight at 4 °C. The corresponding secondary antibody was added and incubated at 37 °C for 30 min. SABC was added and incubated at 37 °C for 30 min; then, the chromogenic agent was added. After color development, the slices were rinsed with water and blotted dry, and the chromogenic agent was added again for counterstaining. After counterstaining, the slices were rinsed with water and successively immersed in 70%, 80%, 90%, 95%, and 100% ethanol and xylene. A drop of neutral balsam was placed on the glass slide; then, a coverslip was placed on it, and the slide was placed in a fume hood. After air-drying, the samples were observed under an inverted microscope.

### 2.8. Dual-Luciferase Reporter Assay

The vector digestion system was prepared according to the kit instructions. The sample was gently pipetted until mixed well and then placed in a water bath at 37 °C for 1–2 h. After the digestion reaction was complete, agarose gel electrophoresis was performed, and the target fragment was recovered. The target gene sequence was synthesized directly onto the vector, and the target fragment was amplified. The target fragment was then connected to the vector. It reacted at 50 °C for 30 min and was then placed on ice for 5 min. We took 50 μL of DH5α competent cells and added 5 μL of the ligation product, followed by incubation on ice for 30 min. Heat shock was performed at 42 °C for 90 s, and then the sample was immediately placed in an ice bath for 3 min. In the laminar flow cabinet, we added 500 μL of LB medium without antibiotics. The tube was inverted 5 times and shake-cultured at 37 °C and 230 rpm for 60 min. The bacterial solution was spread evenly onto the solid plate with the corresponding antibiotic resistance and placed in an incubator at 37 °C for inverted culture for 12 h. PCR identification was performed on the bacterial solution. The positive clones were identified, and the sequencing results were compared. We extracted and purified the plasmid according to the kit instructions. The target plasmid was transfected into 293T cells, and detection was conducted according to the instructions of the dual-luciferase reporter assay kit.

### 2.9. RNA Immunoprecipitation (RIP) Assay

Magnetic racks and magnetic beads were prepared. We added 5 μg of the target antibody and IgG and incubated them for 1 h. Following transfection with miR-15a-5p mimic or miR-15a-5p inhibitor, cells were collected into a 1.5 mL EP tube. RIP lysis buffer, protease inhibitor cocktail, and ribonuclease inhibitor were then added. After centrifugation, 100 μL of the supernatant was transferred to the magnetic bead–antibody complex and then incubated overnight at 4 °C. The magnetic bead–antibody complex was subsequently washed with RIP wash buffer. We then added 150 μL of proteinase K buffer and incubated the sample at 55 °C for 30 min. It was placed on a magnetic rack, and the supernatant was aspirated into a new 1.5 mL EP tube. RIP wash buffer, phenol, chloroform, and isoamyl alcohol were then added. After centrifugation, the upper aqueous phase was transferred to a new EP tube. We added 50 μL of salt solution I, 15 μL of salt solution II, 5 μL of precipitation enhancer, and 850 μL of absolute ethanol, and the supernatant was centrifuged and discarded. The precipitate was air-dried naturally. The precipitate was resuspended in 20 μL of DEPC-treated water, and total RNA extraction, cDNA synthesis, and RT-qPCR experiments were performed.

### 2.10. In Vivo Tumor Xenograft Model

The BALB/c male nude mice used in this study were purchased from Beijing Vital River Laboratory Animal Technology Co. and housed at the Animal Experiment Center of Southeast University with approval from the Ethics Committee. Cell suspensions were injected into the axillae of nude mice (6 mice per group), and tumor growth was observed and measured periodically. Nude mice were euthanized approximately 4 weeks post-injection, and tumors were carefully excised and labeled. Tumor volumes were calculated using the formula V = (a × b^2^)/2, where “a” is the longest diameter, and “b” is the shortest diameter of the tumor. Tumors were immersed separately in tissue preservation solution and paraformaldehyde for subsequent analysis.

### 2.11. Statistical Analysis

Statistical methods were used to analyze differences in gene expression between different groups, namely, ANOVA and Student’s *t*-test. All analyses were conducted using R 4.1.3, SPSS 22, Excel 2019, and GraphPad Prism 10. The R packages used included limma, pheatmap, and ggpubr. Quantitative data are expressed as mean ± SD, and the significance level was set at α = 0.05, with * indicating a significant difference compared with the control group (*: *p* < 0.05; **: *p* < 0.01).

## 3. Results

### 3.1. LncRNA TSPEAR-AS2 Is Overexpressed in GCSCs

**Figure 2:**
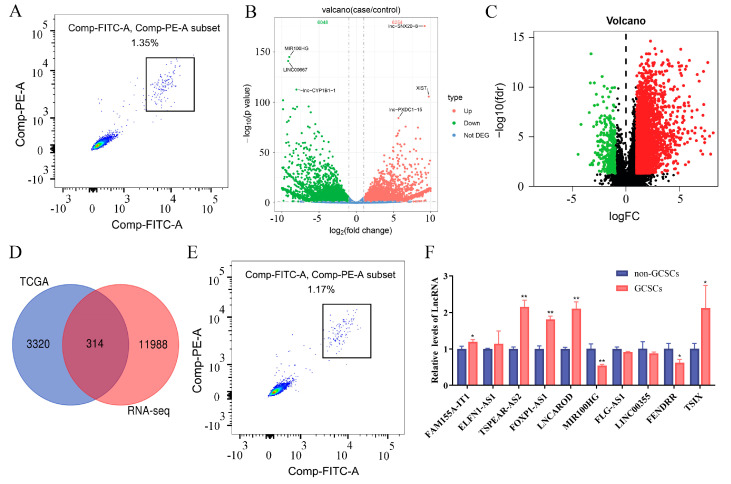
LncRNAs with significant differential expression in GCSCs were analyzed and verified using sequencing and the TCGA database. (**A**) GCSCs were sorted via flow cytometry from gastric tumor tissues. PE indicates CD54 labeling; FITC indicates CD44 labeling; and the box highlights CD54^+^ CD44^+^ cells, identified as GCSCs. (**B**) Volcano plot representing the differentially expressed LncRNAs between non-GCSCs and GCSCs. Red dots indicate upregulation in GCSCs, green dots indicate downregulation in GCSCs, and blue dots indicate no difference in expression between GCSCs and non-GCSCs. (**C**) Volcano plot representing the differentially expressed LncRNAs between normal and GC groups based on the TCGA database. Upregulated and downregulated DEGs are highlighted in red and green, respectively. (**D**) Venn diagrams showing differentially expressed LncRNAs, identifying a total of 314 critical GC stemness-associated LncRNAs. (**E**) GCSCs were sorted by flow cytometry from the GC cell line HGC-27. PE indicates CD54 labeling, FITC indicates CD44 labeling, and the box highlights CD54^+^ CD44^+^ cells, identified as GCSCs. (**F**) Validation of the expression levels of 10 LncRNAs in non-GCSCs and GCSCs. The expression levels of VCAN, FEN1, BRIP1, CNTN1, P3H2, and DUSP1 are shown for non-GCSCs and GCSCs. * *p* < 0.05; ** *p* < 0.01.

GCSCs and non-GCSCs derived from GC tissues were sorted via flow cytometry and subjected to whole-transcriptome sequencing (Figure 2A). Differential expression analysis was performed with DESeq2 under the following criteria: |log_2_(fold change)| > 1 and *p* < 0.05. LncRNAs with log_2_(fold change) > 1 were considered upregulated in GCSCs, while those with log_2_(fold change) < −1 were considered downregulated. The results showed 6254 upregulated and 6048 downregulated LncRNAs in GCSCs compared with non-GCSCs (Figure 2B).

Transcriptome data from 375 GC tissues and 32 adjacent normal tissues were downloaded from The Cancer Genome Atlas (TCGA) database, identifying 3634 differentially expressed LncRNAs using the criteria of *p* < 0.05 and |log_2_(fold change)| > 1. In total, 3334 LncRNAs were upregulated, while 291 were downregulated (Figure 2C).

A Venn diagram was used to identify intersections of differentially expressed LncRNAs from the TCGA database and whole-transcriptome sequencing, revealing 314 LncRNAs that were significantly different between GC tissues and adjacent normal tissues, as well as between GCSCs and non-GCSCs (Figure 2D).

**Figure 3:**
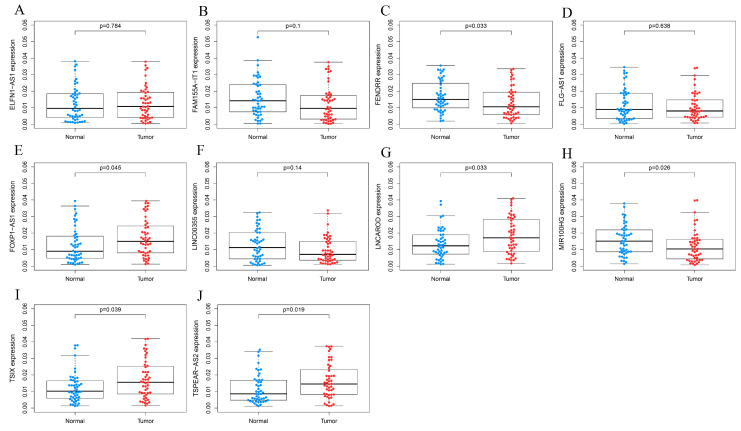
Validation of the expression levels of 10 LncRNAs in tissues. Expression levels of (**A**) ELFN1-AS1, (**B**) FAM155A-IT1, (**C**) FENDRR, (**D**) FLG-AS1, (**E**) FOXP1-AS1, (**F**) LINC00355, (**G**) LNCAROD, (**H**) MIR100HG, (**I**) TSIX, and (**J**) TSPEAR-AS2 in 48 pairs of GC tumor tissues and adjacent non-malignant tissue samples. GC, gastric cancer.

Among these intersecting LncRNAs, those with an average expression value of 0 in any group were excluded. The five most highly expressed and five least expressed LncRNAs were selected as candidates and validated in GC patient tissues. RT-qPCR showed no significant differences in ELFN1-AS1, FLG-AS1, LIN-CO0355, and FAM155A-IT1 expression between tumor and normal tissues (*p* > 0.05). However, FOXP1-AS1, TSIX, INCAROD, FENDRR, MIR100HG, and TSPEAR-AS2 exhibited significantly different expression levels (*p* < 0.05) (Figure 3). Further RT-qPCR confirmed that TSPEAR-AS2 was significantly differentially expressed between GCSCs and non-GCSCs, displaying the highest fold change among the analyzed genes (Figure 2E,F).

### 3.2. LncRNA TSPEAR-AS2 Maintains the Stemness of GCSCs

**Figure 4:**
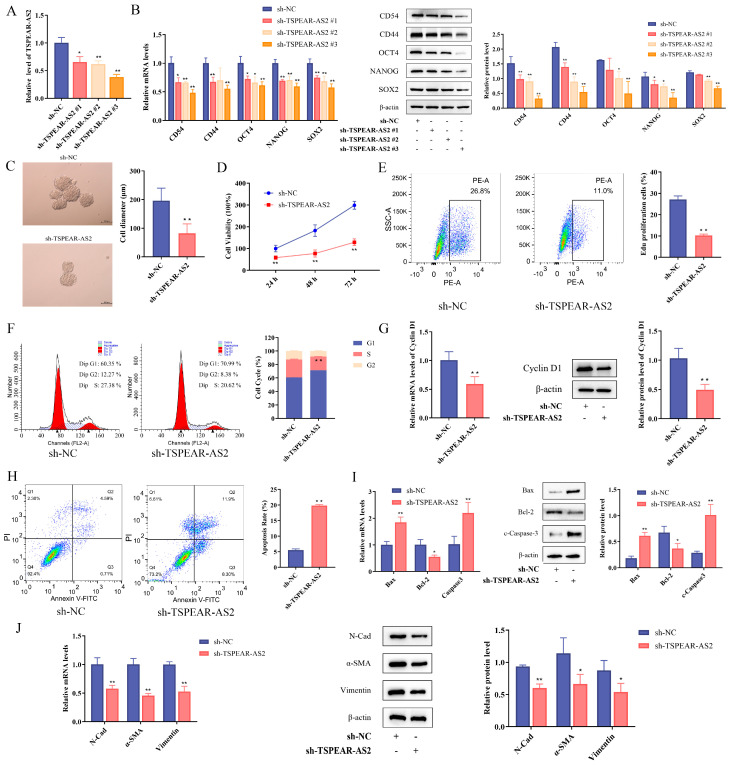
LncRNA TSPEAR-AS2 maintains the stemness of GCSCs. (**A**) Expression levels of TSPEAR-AS2 in GCSCs after transfection. (**B**) Expression of GCSC surface markers and stemness genes was determined by RT-qPCR and Western blot experiments. (**C**) Sphere-forming assay in vitro. Scale bar, 100 μm. (**D**,**E**) Proliferation ability of GCSCs in the TSPEAR-AS2 knockdown group was shown by CCK-8 and EdU assays. (**F**) Cell cycle analysis of GCSCs in the TSPEAR-AS2 knockdown group. (**G**) Expression of Cyclin D1 was determined using RT-qPCR and Western blot experiments. (**H**) Effect of TSPEAR-AS2 expression on GCSC apoptosis was determined using flow cytometry. (**I**) Expression of apoptosis-related genes was determined using RT-qPCR and Western blot experiments. (**J**) Expression of EMT-related genes was determined using RT-qPCR and Western blot experiments. * *p* < 0.05; ** *p* < 0.01. Original images can be found in supplementary materials.

To investigate the role of TSPEAR-AS2 in GCSC stemness, we transfected GCSCs with TSPEAR-AS2 knockdown and overexpression lentiviruses (Figure 4A, Supplementary Figure S1A). The results showed that the expression levels of CD54, CD44, OCT4, NANOG, and SOX2 were significantly downregulated in the TSPEAR-AS2 knockdown group (*p* < 0.05) (Figure 4B) and upregulated in the TSPEAR-AS2 overexpression group (*p* < 0.05) (Supplementary Figure S1B). The lentivirus exhibiting the highest transfection efficiency was selected for subsequent experiments.

In vitro sphere-forming assays showed that both the number and size of spheres were significantly reduced in the TSPEAR-AS2 knockdown group compared with the NC group (Figure 4C), while the opposite results were observed in the overexpression group (Supplementary Figure S1C). A CCK-8 assay showed significantly decreased cell viability in the knockdown group at 24 h, 48 h, and 72 h (*p* < 0.05) (Figure 4D). EdU staining also showed reduced incorporation in the knockdown group (*p* < 0.05) (Figure 4E), while proliferation was enhanced in the overexpression group (Supplementary Figure S1D,E). Cell cycle analysis revealed G1 phase arrest in the TSPEAR-AS2 knockdown group (*p* < 0.05) (Figure 4F). Both transcription and translation of Cyclin D1 were reduced in the TSPEAR-AS2 knockdown group (*p* < 0.05) (Figure 4G). By contrast, overexpression led to increased Cyclin D1 levels and S-phase arrest (*p* < 0.05) (Supplementary Figure S1F,G). Flow cytometry analysis showed a higher apoptosis rate in the TSPEAR-AS2 knockdown group (*p* < 0.05) (Figure 4H). Bax and c-Caspase3 expression were increased, while Bcl2 expression showed a decreasing trend (*p* < 0.05) (Figure 4I). Furthermore, TSPEAR-AS2 knockdown promoted EMT in GCSCs, as shown by reduced N-Cadherin, α-SMA, and Vimentin expression (*p* < 0.05) (Figure 4J). The overexpression group exhibited opposite trends (Supplementary Figure S1H–J). Collectively, these findings suggest that TSPEAR-AS2 plays a key role in maintaining GCSC stemness.

### 3.3. TSPEAR-AS2 Accelerated Cell Growth of GCSCs In Vivo

**Figure 5:**
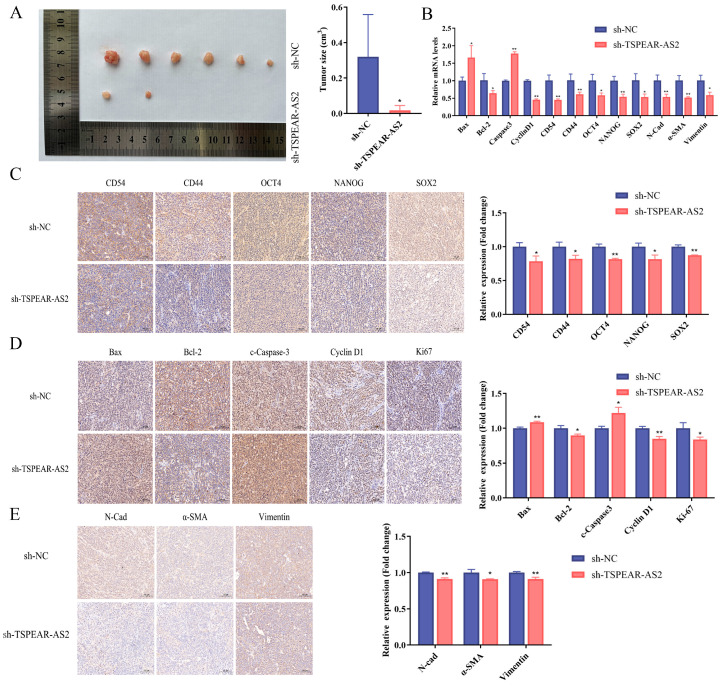
Validation of the regulatory effect of TSPEAR-AS2 on GCSCs through subcutaneous tumor formation experiments on nude mice. (**A**) Volume of subcutaneous tumors in nude mice. (**B**) Expression of CD54, CD44, OCT4, NANOG, SOX2, Cyclin D1, Bcl2, Caspase3, Bax, N-Cad, α-SMA, and Vimentin in excised tumors was determined using RT-qPCR. (**C**) Validation of stemness-associated protein expression in tumor tissues using immunohistochemistry. Scale bar, 100 μm. (**D**) Validation of cell cycle, apoptosis, and proliferation-related protein expression in tumor tissues. Scale bar, 100 μm. (**E**) Validation of EMT-related protein expression in tumor tissues. Scale bar, 100 μm. * *p* < 0.05; ** *p* < 0.01.

To determine whether TSPEAR-AS2 regulates GCSCs in vivo, we performed subcutaneous tumor formation assays on nude mice. The results showed that nude mice injected with TSPEAR-AS2 knockdown cells developed significantly smaller tumors than the NC group (Figure 5A), while those injected with TSPEAR-AS2-overexpressing cells developed larger tumors (Supplementary Figure S2A). The expression levels of CD54, CD44, OCT4, NANOG, and SOX2 were significantly reduced in the TSPEAR-AS2 knockdown group compared with the NC group (*p* < 0.05). Bcl2, Cyclin D1, N-Cadherin, α-SMA, and Vimentin were all downregulated, while Caspase3 and Bax expression were upregulated (*p* < 0.05) (Figure 5B). All of these genes exhibited opposite expression patterns in the TSPEAR-AS2 overexpression group (Supplementary Figure S2B).

We further performed immunohistochemistry to assess protein expression in the tumor tissues. TSPEAR-AS2 knockdown significantly reduced CD54, CD44, OCT4, NANOG, and SOX2 levels (*p* < 0.05) (Figure 5C), indicating reduced GCSC stemness. Bax and c-Caspase3 increased, while anti-apoptotic protein Bcl2 decreased (*p* < 0.05) (Figure 5D), suggesting that TSPEAR-AS2 knockdown enhances apoptosis in GCSCs. Cyclin D1 was also downregulated (*p* < 0.05) (Figure 5D), suggesting that low TSPEAR-AS2 expression may lead to cell cycle arrest in GCSCs. Ki67 expression was significantly decreased (*p* < 0.05) (Figure 5D), suggesting reduced GCSCs proliferation. N-Cadherin, α-SMA, and Vimentin were also reduced (*p* < 0.05) (Figure 5E), suggesting the suppression of the EMT process. Overexpression group experiments showed opposite trends (Supplementary Figure S2C–E).

### 3.4. TSPEAR-AS2 Directly Targeted miR-15a-5p in GCSCs

**Figure 6:**
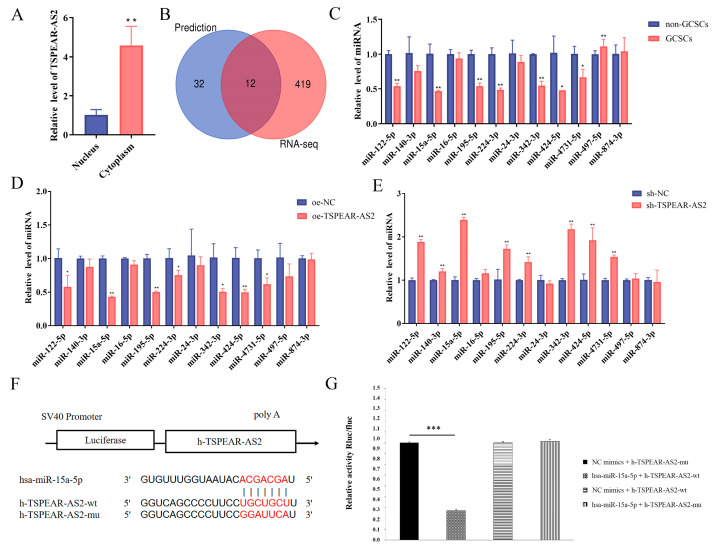
Prediction and validation of TSPEAR-AS2-associated miRNA. (**A**) Cellular localization of TSPEAR-AS2. (**B**) Screening and validation of miRNAs associated with TSPEAR-AS2. (**C**) Expression of miRNAs in GCSCs and non-GCSCs was determined using RT-qPCR. (**D**) Effect of TSPEAR-AS2 overexpression on miR-15a-5p expression in GCSCs. (**E**) Effect of TSPEAR-AS2 knockdown on miR-15a-5p expression in GCSCs. (**F**) Schematic representation of miR-15a-5p binding to TSPEAR-AS2 target sites. (**G**) Dual-luciferase reporter assay to detect the interaction between miR-15a-5p and TSPEAR-AS2. * *p* < 0.05; ** *p* < 0.01; *** *p* < 0.001.

LncRNAs can regulate CSC functions by acting as molecular sponges for downstream miRNAs. To identify miRNAs targeted by TSPEAR-AS2, we first performed a nucleoplasmic isolation assay and determined that TSPEAR-AS2 was mainly localized in the cytoplasm, suggesting its potential as a miRNA molecular sponge (Figure 6A). TSPEAR-AS2-related miRNAs were predicted using bioinformatics methods, yielding 44 miRNAs. Further, 12 candidate miRNAs were identified by intersecting these with 431 miRNAs that were downregulated in the whole-transcriptome sequencing results (Figure 6B). RT-qPCR showed that miR-497-5p was significantly upregulated in GCSCs, while miR-122-5p, miR-15a-5p, miR-195-5p, miR-224-3p, miR-342-3p, miR-424-5p, and miR-4731-5p were downregulated in GCSCs compared with non-GCSCs (*p* < 0.05) (Figure 6C). Of these, miR-15a-5p was expressed at the lowest level in GCSCs with TSPEAR-AS2 overexpression, showing the highest fold change (*p* < 0.05) (Figure 6D). Conversely, miR-15a-5p expression was significantly upregulated in TSPEAR-AS2 knockdown cells (*p* < 0.05) (Figure 6E). Thus, miR-15a-5p was selected for subsequent studies.

We further examined the binding interactions between miR-15a-5p and TSPEAR-AS2 using a dual-luciferase reporter assay. Sequence analysis identified miR-15a-5p binding sites within the TSPEAR-AS2 sequence (Figure 6F). miR-15a-5p significantly reduced the luciferase activity of the wild-type TSPEAR-AS2 construct compared with the control (*p* < 0.05). However, this effect was abolished in the mutant construct (*p* > 0.05) (Figure 6G) (Supplementary Table S2), confirming a direct interaction between miR-15a-5p and TSPEAR-AS2.

### 3.5. miR-15a-5p Inhibited GCSC Stemness

**Figure 7:**
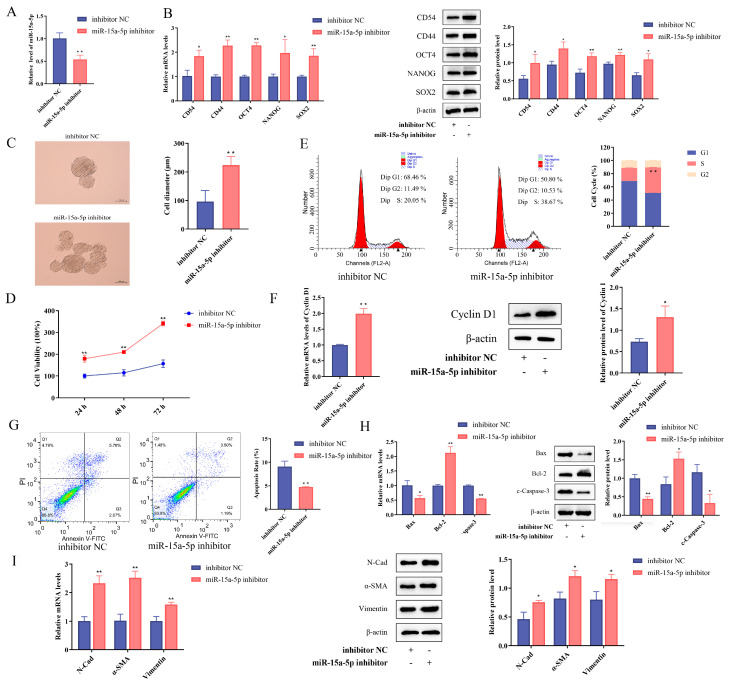
miR-15a-5p inhibits the stemness of GCSCs. (**A**) Expression levels of miR-15a-5p in GCSCs after transfection. (**B**) Expression of GCSC surface markers and stemness genes was determined using RT-qPCR and Western blot experiments. (**C**) Sphere-forming assay in vitro. Scale bar, 100 μm. (**D**) Effect of miR-15a-5p on the proliferation capacity of GCSCs was examined using the CCK-8 assay. (**E**) Cell cycle experiments were performed to explore the effect of miR-15a-5p on the cell cycle of GCSCs. (**F**) Expression of Cyclin D1 was determined using RT-qPCR and Western blot experiments. (**G**) Effect of miR-15a-5p expression on GCSC apoptosis was determined using flow cytometry. (**H**) Expression of apoptosis-related genes was determined using RT-qPCR and Western blot experiments. (**I**) Expression of EMT-related genes was determined using RT-qPCR and Western blot experiments. * *p* < 0.05; ** *p* < 0.01. Original images can be found in supplementary materials.

To investigate the function of miR-15a-5p, GCSCs were transfected with miR-15a-5p mimics or a miR-15a-5p inhibitor (Figure 7A) (Supplementary Figure S3A). RT-qPCR and Western blot showed that miR-15a-5p overexpression inhibited the expression of surface markers and stemness genes in GCSCs (Figure 7B), while inhibition enhanced stemness (Supplementary Figure S3B). The sphere-forming assay demonstrated that miR-15a-5p overexpression inhibited the sphere-forming ability of GCSCs (Figure 7C), whereas knockdown had the opposite effect (Supplementary S3C).

In addition, overexpression of miR-15a-5p inhibited GCSC proliferation (Figure 7D) and induced G1 phase arrest (Figure 7E), accompanied by reduced Cyclin D1 expression at both mRNA and protein levels (Figure 7F). Flow cytometry showed a higher apoptosis rate in the overexpression group than in the NC group (*p* < 0.05) (Figure 7G). RT-qPCR and Western blot further revealed increased Bax and Caspase3 expression and decreased Bcl2 in the overexpression group (Figure 7H), indicating enhanced apoptosis. N-Cadherin, α-SMA, and Vimentin were downregulated (Figure 7I), suggesting EMT suppression. Opposite trends were observed in the knockdown group (Supplementary Figure S3D–I). Collectively, these results indicate that miR-15a-5p can regulate the stemness of GCSCs.

### 3.6. TSPEAR-AS2 Sponged miR-15a-5p to Regulate the Stemness of GCSCs

**Figure 8:**
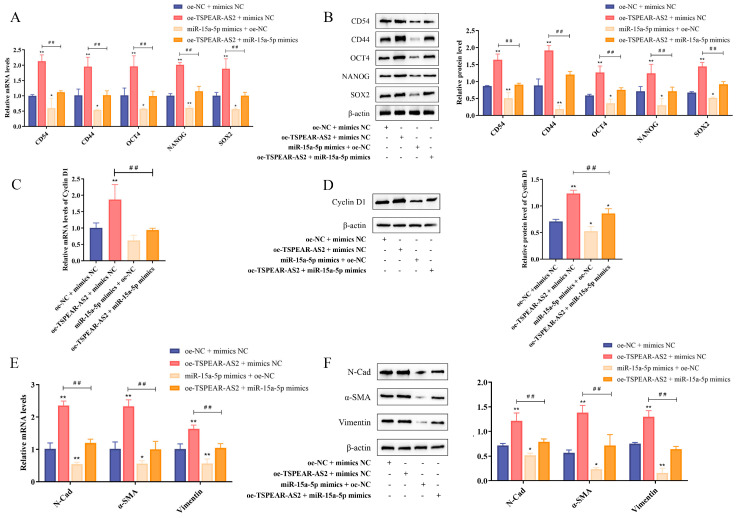
TSPEAR-AS2 sponges miR-15a-5p to regulate the stemness of GCSCs. Expression of GCSC surface markers and stemness genes was determined using (**A**) RT-qPCR and (**B**) Western blot experiments. Expression of Cyclin D1 was determined using (**C**) RT-qPCR and (**D**) Western blot experiments. Expression of EMT-related genes was determined using (**E**) RT-qPCR and (**F**) Western blot experiments. * *p* < 0.05; ** *p* < 0.01; ^##^ *p* < 0.01. Original images can be found in supplementary materials.

Previous studies have shown that miR-15a-5p regulates GCSC stemness and shows an inverse expression pattern compared with TSPEAR-AS2. To explore their relationship, we conducted a rescue assay by co-transfecting TSPEAR-AS2 overexpression lentivirus and miR-15a-5p mimics into GCSCs. This included the NC group (oe-NC + mimics NC), the oe-TSPEAR-AS2 group (oe-TSPEAR-AS2 + mimics NC), the miR-15a-5p mimics group (oe-NC + miR-15a-5p mimics), and a co-transfection group (oe-TSPEAR-AS2 + miR-15a-5p mimics). The results showed that CD54, CD44, OCT4, NANOG, and SOX2 significantly decreased in the co-transfection group compared with the oe-TSPEAR-AS2 group (*p* < 0.05) (Figure 8A,B), indicating that miR-15a-5p overexpression partially reverses the effect of TSPEAR-AS2 overexpression on GCSC stemness. The transcription and translation levels of Cyclin D1 in the co-transfection group were significantly lower than those in the TSPEAR-AS2 overexpression group (Figure 8C,D), indicating that miR-15a-5p can counteract TSPEAR-AS2′s effect on the cell cycle. Compared with the oe-TSPEAR-AS2 group, N-Cadherin, α-SMA, and Vimentin expression was downregulated in the co-transfected group (*p* < 0.05), with no significant difference compared with the NC group (Figure 8E,F), indicating that miR-15a-5p overexpression reverses TSPEAR-AS2′s effect on EMT.

We further tested whether the effects of TSPEAR-AS2 knockdown on GCSCs could also be reversed by miR-15a-5p. The experimental groups included the NC group (sh-NC + inhibitor NC), the sh-TSPEAR-AS2 group (sh-TSPEAR-AS2 + inhibitor NC), the miR-15a-5p inhibitor group (sh-NC + miR-15a-5p inhibitor), and the co-transfection group (sh-TSPEAR-AS2 + miR-15a-5p inhibitor). RT-qPCR and Western blot analyses showed that CD54, CD44, OCT4, NANOG, and SOX2 were higher in the co-transfection group than in the TSPEAR-AS2 knockdown group (*p* < 0.05) (Supplementary Figure S4A). Cyclin D1 expression was elevated in the co-transfection group (*p* < 0.05) (Supplementary Figure S4B). N-Cadherin, α-SMA, and Vimentin levels also increased in the co-transfection group (*p* < 0.05) (Supplementary Figure S4C), suggesting that miR-15a-5p inhibition partially counteracts the effects of TSPEAR-AS2 knockdown on EMT. These data indicate that TSPEAR-AS2 interacts with miR-15a-5p to regulate the stemness of GCSCs.

### 3.7. CCND1 Is a Direct Target of miR-15a-5p

**Figure 9:**
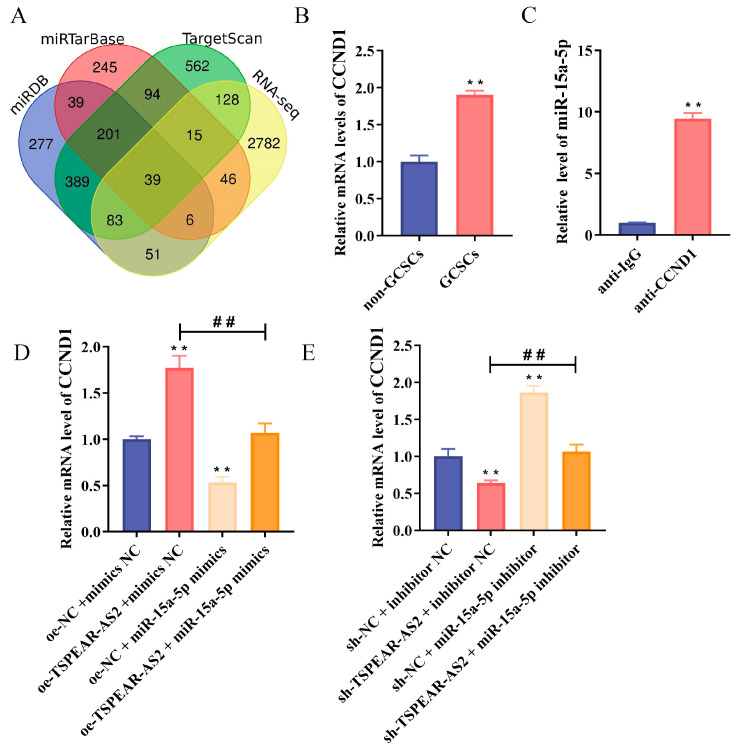
Prediction and validation of miR-15a-5p target genes. (**A**) The miR-15a-5p target genes were predicted using a bioinformatics approach. (**B**) CCND1 expression in GCSCs and non-GCSCs was analyzed. (**C**) The interaction between miR-15a-5p and CCND1 was examined using the RIP assay. (**D**) and (**E**) demonstrate that TSPEAR-AS2 regulates CCND1 expression via miR-15a-5p. ** *p* < 0.01; ^##^ *p* < 0.01.

The miRDB, miRTarBase, and TargetScan databases were used to predict miR-15a-5p-related target genes, ultimately identifying 240 target genes. By intersecting these with upregulated genes from whole-transcriptome sequencing, we obtained 39 miR-15a-5p-related target genes (Figure 9A). RT-qPCR showed that CCND1 expression was significantly upregulated in GCSCs compared with non-GCSCs (*p* < 0.05) (Figure 9B). To investigate the binding relationship between miR-15a-5p and CCND1, we conducted an RIP assay. The results showed that miR-15a-5p was successfully amplified from CCND1 immunoprecipitation complexes, and its expression level was significantly higher than in the control group (*p* < 0.05) (Figure 9C), suggesting that miR-15a-5p directly binds to CCND1. To determine whether TSPEAR-AS2 regulates CCND1 via miR-15a-5p, we used a rescue assay. The experimental groups included the NC group (oe-NC + mimics NC), the oe-TSPEAR-AS2 group (oe-TSPEAR-AS2 + mimics NC), the miR-15a-5p mimics group (oe-NC + miR-15a-5p mimics), and the co-transfection group (oe-TSPEAR-AS2 + miR-15a-5p mimics). RT-qPCR showed that TSPEAR-AS2 overexpression upregulated CCND1 expression compared with the NC group (*p* < 0.05), while miR-15a-5p overexpression reduced CCND1 expression (*p* < 0.05). No significant difference was observed between the co-transfection group and the NC group. CCND1 expression was significantly higher in the oe-TSPEAR-AS2 group than in the co-transfection group (*p* < 0.05), suggesting that miR-15a-5p reverses the effect of TSPEAR-AS2 on CCND1 (Figure 9D).

Similarly, the rescue assay was performed using an interfering lentivirus, with the following groups: the NC group (sh-NC + inhibitor NC), the sh-TSPEAR-AS2 group (sh-TSPEAR-AS2 + inhibitor NC), the miR-15a-5p inhibitor group (sh-NC + miR-15a-5p inhibitor), and the co-transfection group (sh-TSPEAR-AS2 + miR-15a-5p inhibitor). The RT-qPCR results showed that CCND1 expression in the sh-TSPEAR-AS2 group was decreased compared with the NC group (*p* < 0.05) and increased in the miR-15a-5p inhibitor group (*p* < 0.05). No significant change was observed in the co-transfection group. Moreover, CCND1 expression in the sh-TSPEAR-AS2 group was significantly lower than in the co-transfection group (*p* < 0.05), suggesting that the miR-15a-5p inhibitor could restore CCND1 expression in the sh-TSPEAR-AS2 group (*p* < 0.05) (Figure 9E).

## 4. Discussion

The prevalence of GC remains high, and the prognosis for advanced GC patients is poor, with limited treatment options that severely compromise quality of life [34,35]. In recent years, researchers have actively sought novel therapeutic targets to improve the efficacy of GC treatment and enhance patient outcomes. CSCs have emerged as a key focus of research and are increasingly recognized as a primary source of tumor initiation and progression [36,37]. Thus, targeting CSCs represents a promising strategy for effective cancer control [38].

Previous studies have demonstrated that LncRNAs play a critical role in regulating CSCs. For example, LncRNA PVT1 enhances the sphere-forming ability of nasopharyngeal cancer CSCs [39], and LncRNA HOTTIP promotes tumorigenicity and spheroid formation in pancreatic CSCs, as shown by both in vitro and in vivo experiments [40]. Additionally, LncRNA FMRI-AS1 has been reported to enhance the proliferative capacity of esophageal CSCs [41]. Building on these findings, we screened for LncRNAs associated with GCSCs and identified LncRNA TSPEAR-AS2 as a promising candidate. Studies have shown that TSPEAR-AS2 is involved in regulating multiple cancers. Peng et al. found that TSPEAR-AS2 regulates fatty acid metabolism in colorectal cancer patients [42]. In oral squamous cell carcinoma, TSPEAR-AS2 facilitates tumor progression by competitively inhibiting miR-487a-3p and modulating its target gene, PPM1A44 [43]. Moreover, in gastric cancer, TSPEAR-AS2 promotes disease progression by upregulating CLDN4 via miR-1207-5p [44]. Despite these findings, the role of TSPEAR-AS2 in GCSCs remains underexplored, and its underlying mechanisms are not yet fully understood.

In this study, we established stable cell models with TSPEAR-AS2 knockdown and overexpression. After knocking down TSPEAR-AS2, the expression levels of CD54, CD44, Oct4, Nanog, and Sox2 significantly decreased, while overexpressing TSPEAR-AS2 significantly upregulated the expression of these genes. This result demonstrated the effect of TSPEAR-AS2 on the stemness of gastric cancer stem cells (GCSCs) at the gene expression level.

Sphere-forming capacity is an important manifestation of the stemness characteristics of CSCs [45]. The in vitro sphere-forming assay uses serum-free culture to isolate cells with sphere-forming abilities from candidate cells. These cells are considered to have a self-renewal ability, and the strength of this ability can be demonstrated by the number of spheres formed. Accumulating evidence indicates that LncRNAs regulate the sphere-forming capacity of CSCs. For instance, LncRNA MEG3 downregulation enhances sphere formation and CSC properties [46]. In this study, we demonstrated that TSPEAR-AS2 overexpression promotes the spheroid-forming and tumor-forming abilities of GCSCs, whereas its knockdown suppresses these functions.

CSCs possess the proliferative potential to regenerate and disseminate metastatic tumors. Alterations in the stemness-related functions of CSCs are often accompanied by corresponding changes in their proliferative capacity [46]. For example, LncRNA SNHG5 promotes proliferation and other stemness-related functions in hepatocellular carcinoma stem cells [47]. Similarly, we found that TSPEAR-AS2 overexpression and knockdown increase and decrease the proliferative ability of GCSCs, respectively.

Alterations in the cell cycle and apoptotic processes are critical for the abnormal biological behavior of cancer stem cells. Apoptosis is a mode of cell death that plays a crucial role in tumor formation, and LncRNA knockdown WDFY3-AS2 induces apoptosis in OC stem cells [48]. As a recognized oncogene, silencing LncRNA TUG1 facilitates apoptosis in CRC stem cells [49]. In our study, a decrease in TSPEAR-AS2 expression promoted apoptosis in GCSCs, while overexpression inhibited it. Detection of apoptosis-related genes showed that downregulating TSPEAR-AS2 expression increased the expression of Bax and Caspase3 and inhibited the expression of Bcl-2. Bax is a major pro-apoptotic member of the Bcl-2 family. It can form a multimer with Bcl-2, enhance mitochondrial permeability, lead to the release of cytochrome C, and promote apoptosis. Bcl-2 is an anti-apoptotic member and can inhibit the action of the former or the release of mitochondrial cytochrome C. Caspase3 is a downstream effector cysteine protease in the apoptotic pathway and plays a crucial role in apoptosis. Its abnormal activation is present in various malignant tumors. Therefore, we hypothesized that TSPEAR-AS2 may maintain the anti-apoptotic characteristics of GCSCs by regulating the inhibition of Caspase3 by Bcl-2 and suppressing the cytotoxicity of Bax. Further in-depth research on this apoptotic pathway is needed.

The cell cycle is a fundamental process of life activities and is regulated by multiple factors. Regulating cell cycle progression has been suggested as a promising approach to controlling tumor growth. The cell cycle includes the G1, S, and G2 phases of DNA synthesis, followed by the M phase [50]. In this study, we found that aberrant expression of TSPEAR-AS2 alters the cell cycle in GC stem cells. Flow cytometry analysis showed that GCSCs with knocked-down TSPEAR-AS2 were arrested in the G1 phase, and the expression of Cyclin D1 decreased. After overexpressing TSPEAR-AS2, the proportion of cells in the S phase increased, and the expression of Cyclin D1 was significantly elevated. The DNA content in cells during the S and G2/M phases of the cell cycle is more than twice that of G0/G1 phase cells. The more cells are in these two phases during the process of entering the next round of division, the more active cell proliferation is. Cyclins are proteins whose concentrations increase and decrease synchronously with the cell cycle of eukaryotic cells. They include proteins A, B, D, E, G, and H, and Cyclin D1 is an important member among them, indirectly reflecting the progress of the cell cycle. These results indicate that TSPEAR-AS2 can affect the proliferation of GCSCs by altering the cell cycle progression of GCSCs.

EMT is a well-established mechanism underlying CSC biology [36]. It is currently widely believed that cancers originating from epithelia are determined by the EMT process. After undergoing EMT, tumor cells can acquire stem cell-like characteristics. The activation of EMT is associated with the generation of CSCs, and there is a connection between EMT, stemness, and the metastatic initiation potential of tumor cells. However, it remains unclear whether EMT is involved in the process by which TSPEAR-AS2 affects the stemness characteristics of GCSCs. Therefore, in this study, we initially explored this by detecting the expression of EMT-related genes through RT-qPCR and Western blot experiments. The results showed that after knocking down TSPEAR-AS2, the expression levels of the N-cadherin, α-SMA, and Vimentin genes significantly decreased, while overexpressing TSPEAR-AS2 significantly upregulated the expression of these genes. This indicates that maintaining GCSC stemness with TSPEAR-AS2 may involve the EMT process. However, the specific regulatory pathways and mechanisms remain unclear and require more experimental research to be revealed.

Emerging evidence indicates that the LncRNA-miRNA-mRNA regulatory pathway is a critical mechanism through which LncRNAs influence the function of CSCs. LncRNAs competitively bind to downstream miRNAs via the ceRNA mechanism, thereby modulating miRNA expression levels, which subsequently regulate cancer by influencing the expression of corresponding target genes. This regulatory mechanism has been widely observed in LncRNA-mediated control of CSCs. For example, LncRNA H19 promotes the proliferation and self-renewal of breast CSCs by sequestering miR-Let-7c and upregulating targets in the estrogen receptor 1 and Wnt signaling pathways. Both knockdown of LncRNA H19 and upregulation of miR-Let-7c impair the stemness-associated functions of breast CSCs [51]. LncRNA XIST inhibits miR-200c expression, thereby modulating the tumorigenic capacity of bladder CSCs [52]. DGCR5 acts as a key regulator of CSCs in non-small-cell carcinoma by modulating the miR-330-5p/CD44 axis [53]. Additionally, miR-124 interacts with LncRNA MALAT1 and is involved in regulating HBx-induced CSC properties via the PI3K/Akt signaling pathway [54].

## 5. Conclusions

In this study, we demonstrated that TSPEAR-AS2 interacts with miR-15a-5p via a ceRNA mechanism, as predicted by bioinformatics analysis, and we validated this relationship using the rescue assay. In both in vitro and in vivo models, Shen et al. showed that miR-15a-5p regulates GCSC stemness by targeting ONECUT2 [55]. We further predicted that miR-15a-5p may regulate GCSCs by modulating the expression of CCND1. Consistent with this hypothesis, knockdown and overexpression of TSPEAR-AS2 inhibited and promoted CCND1 expression, respectively. Similarly, knockdown and overexpression of miR-15a-5p promoted and repressed CCND1 expression, respectively, indicating that the regulatory effect of TSPEAR-AS2 on CCND1 was mediated by miR-15a-5p. Taken together, these findings suggest that TSPEAR-AS2 exerts its regulatory effects on GCSCs via the TSPEAR-AS2/miR-15a-5p/CCND1 axis.

Notably, CCND1, also known as Cyclin D1, is a cell cycle regulatory gene that we have previously studied, suggesting that TSPEAR-AS2 may influence the cell cycle and thus regulate GCSCs through the miR-15a-5p/CCND1 axis. In conclusion, our experiments verified that TSPEAR-AS2 regulates GCSC stem cell properties through the miR-15a-5p/CCND1 axis, highlighting its potential as a novel therapeutic biomarker for GC therapy.

## Data Availability

The whole-transcriptome sequencing data are available on the GEO database under accession number GSE274738.

## Supplementary Materials

The following supporting information can be downloaded at https://www.mdpi.com/article/10.3390/biom15091227/s1. Figure S1: TSPEAR-AS2 overexpression enhanced stemness of GCSCs. Figure S2: The effect of TSPEAR-AS2 overexpression on GCSCs through subcutaneous tumor formation experiments. Figure S3: miR-15a-5p overexpression inhibited the stemness of GCSCs. Figure S4: TSPEAR-AS2 regulates the stemness of GCSCs by binding to miR-15a-5p. Table S1. The Primers of Genes. Table S2. Detection of miR-15a-5p and TSPEAR-AS2 luciferase activity.

## Abbreviations

The following abbreviations are used in this manuscript:

CSCs	cancer stem cells
ceRNA	competing endogenous RNA
EMT	epithelial–mesenchymal transition
GC	gastric cancer
GCSCs	gastric cancer stem cells
GEO	Gene Expression Omnibus
IARC	International Agency for Research on Cancer
IHC	immunohistochemistry
LncRNAs	long non-coding RNAs
RIP	RNA immunoprecipitation
RT-qPCR	quantitative real-time polymerase chain reaction
TCGA	The Cancer Genome Atlas
