# Supplementary material for: LncRNA TSPEAR-AS2 Maintains the Stemness of Gastric Cancer Stem Cells by Regulating the miR-15a-5p/CCND1 Axis
Source: Biomolecules. 2025 Aug 26;15(9):1227. doi: 10.3390/biom15091227 (PMC12467362; doi:10.3390/biom15091227)

Figure 4B

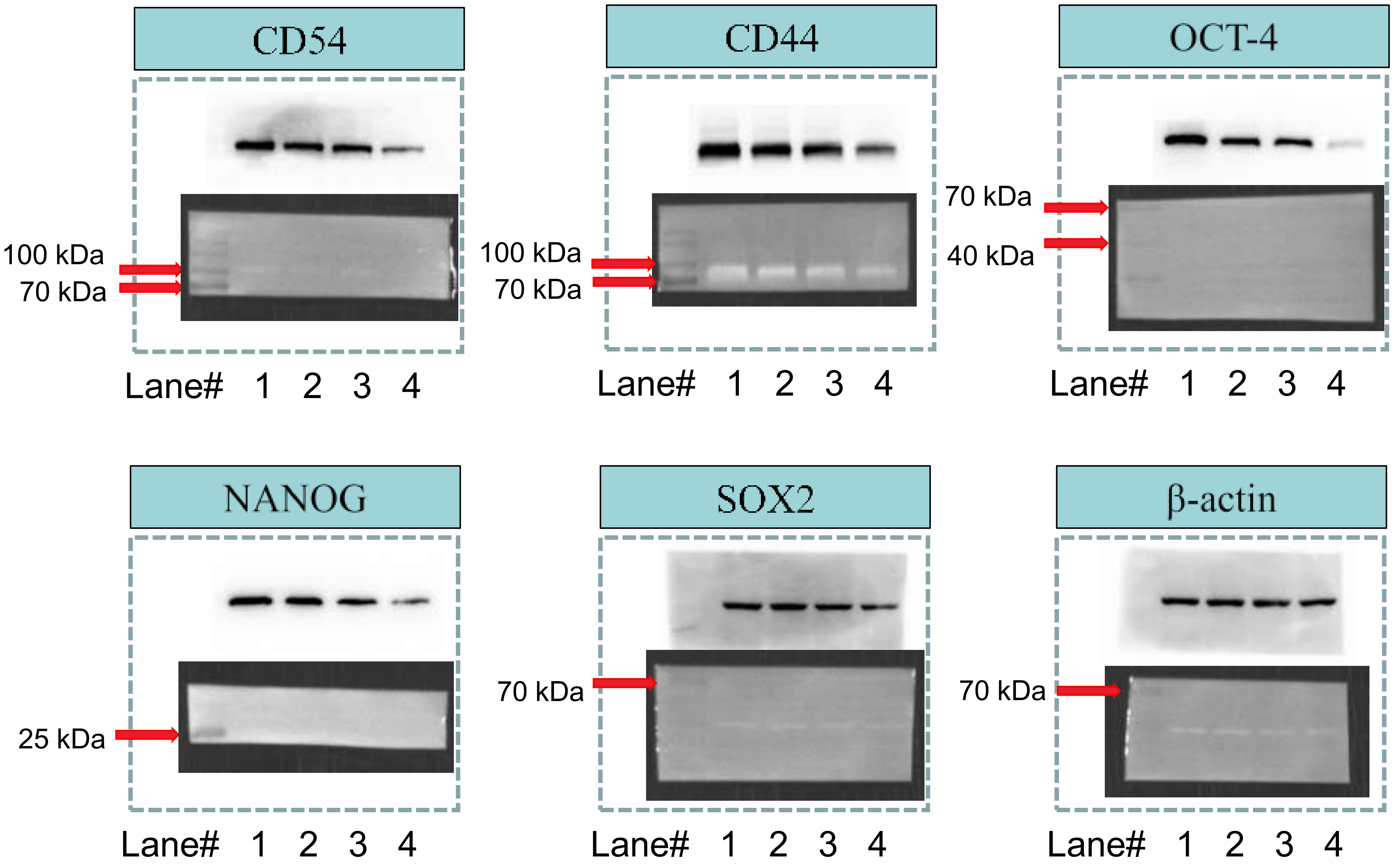
CD54
100 kDa
70 kDa
Lane# 1 2 3 4
CD44
100 kDa
70 kDa
Lane# 1 2 3 4
OCT-4
70 kDa
40 kDa
Lane# 1 2 3 4
NANOG
25 kDa
Lane# 1 2 3 4
SOX2
70 kDa
Lane# 1 2 3 4
β-actin
70 kDa
Lane# 1 2 3 4

Figure 4G

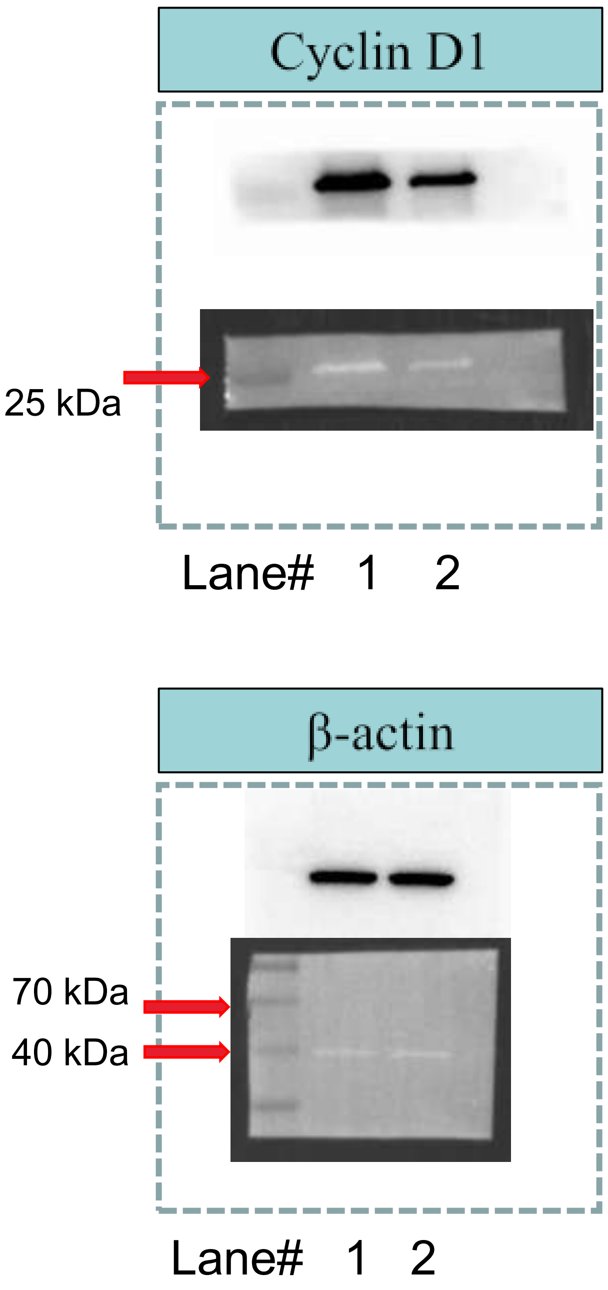

Figure 4I

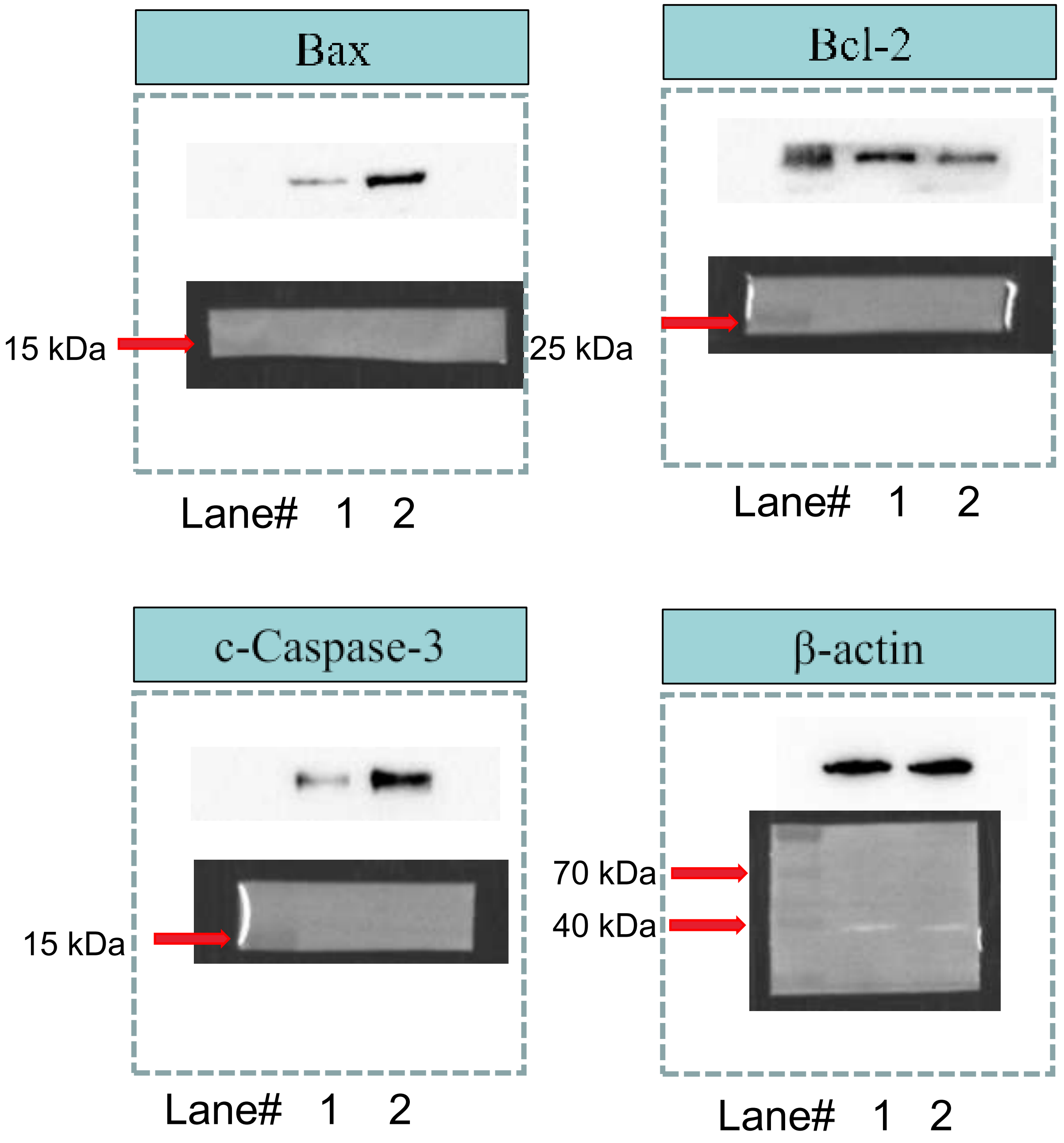

Figure 4J

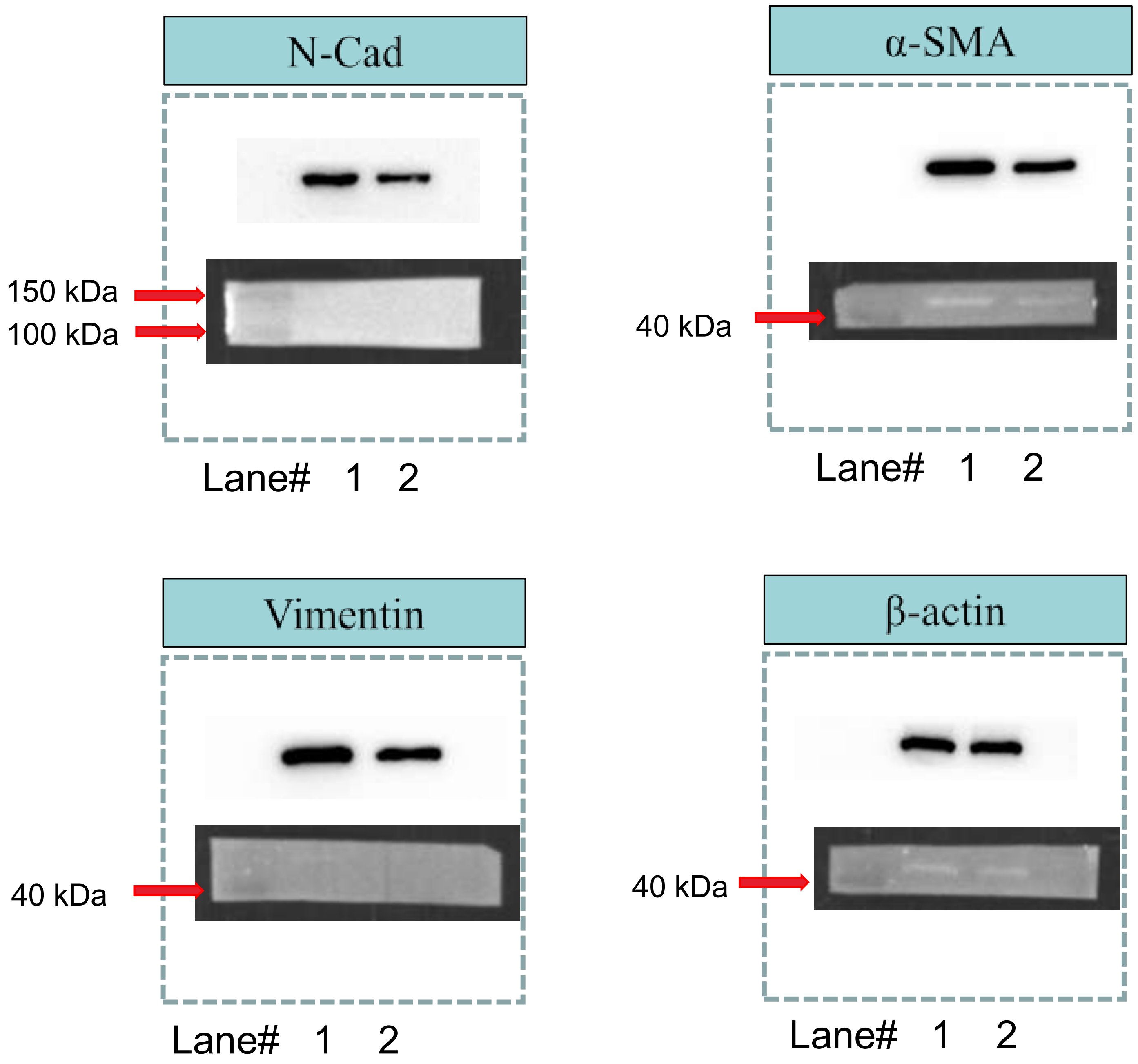
N-Cad
150 kDa
100 kDa
Lane# 1 2
α-SMA
40 kDa
Lane# 1 2
Vimentin
40 kDa
Lane# 1 2
β-actin
40 kDa
Lane# 1 2

Figure 7B

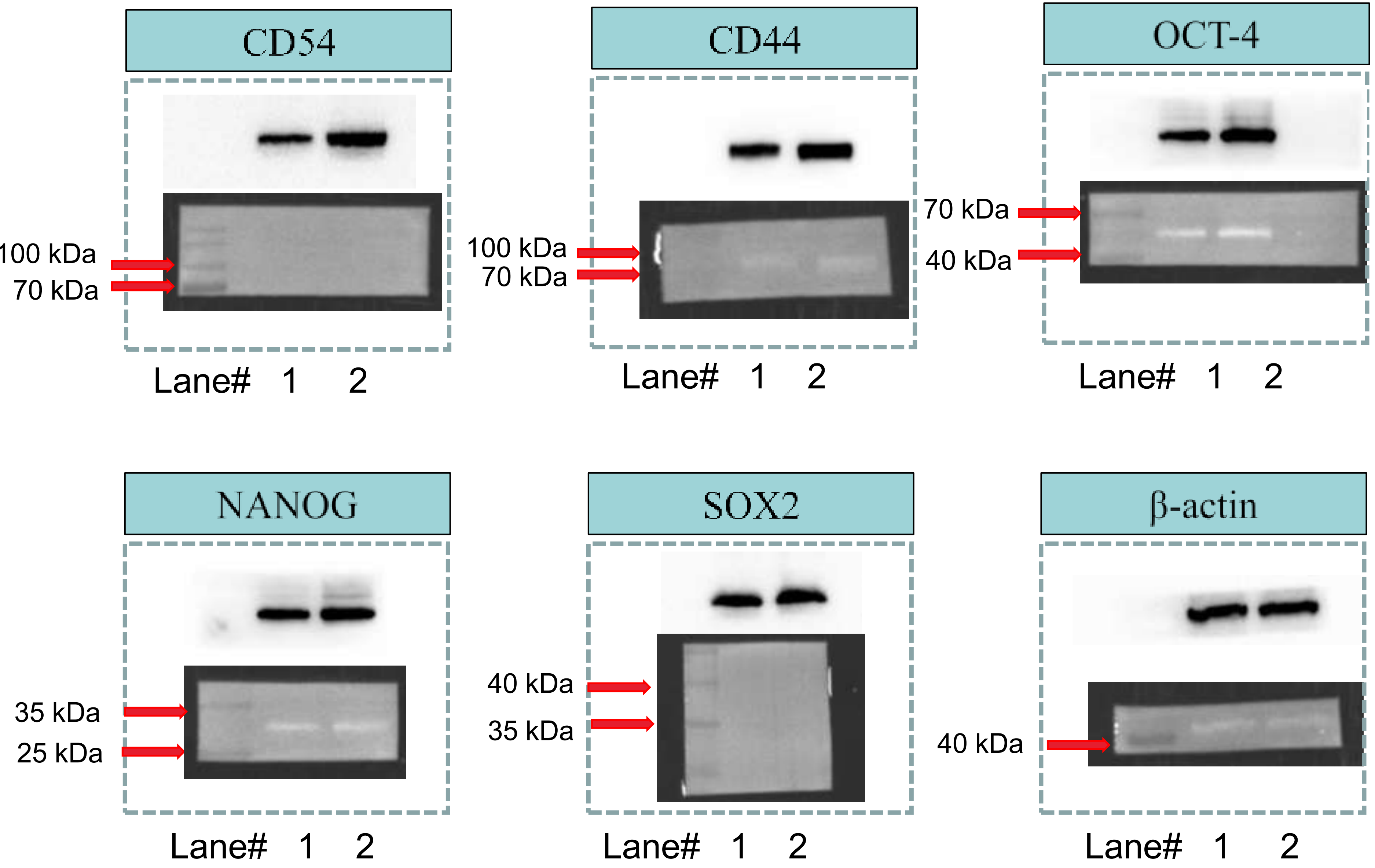
CD54
100 kDa
70 kDa
Lane# 1 2
CD44
100 kDa
70 kDa
Lane# 1 2
OCT-4
70 kDa
40 kDa
Lane# 1 2
NANOG
35 kDa
25 kDa
Lane# 1 2
SOX2
40 kDa
35 kDa
Lane# 1 2
β-actin
40 kDa
Lane# 1 2

Figure 7F

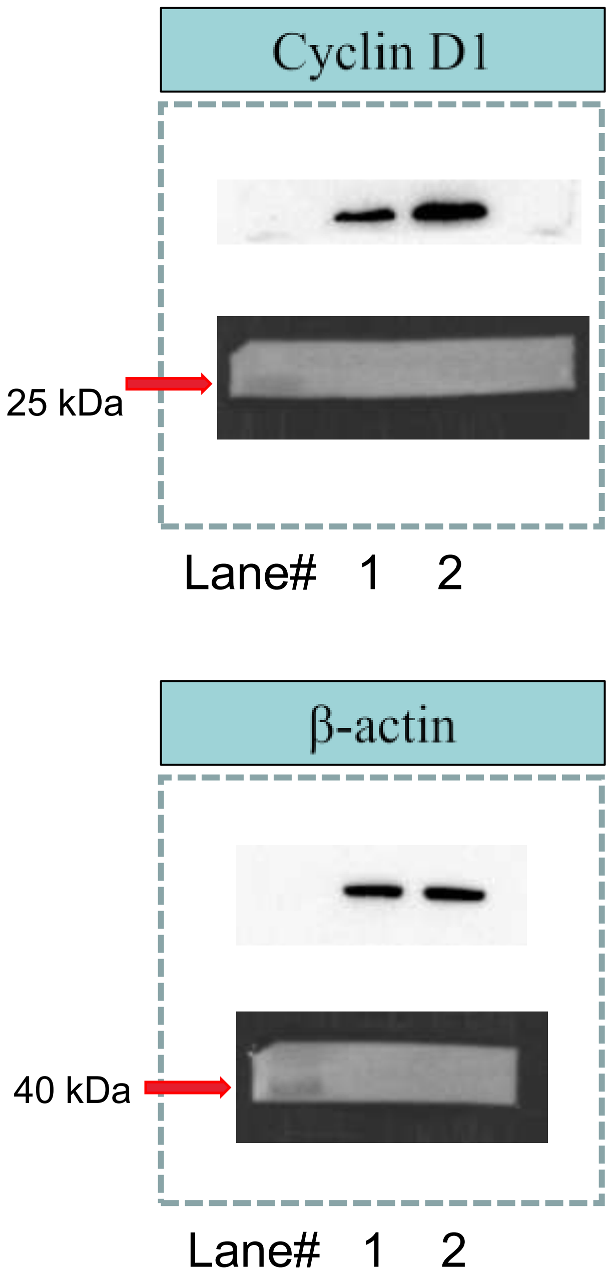

Figure 7H

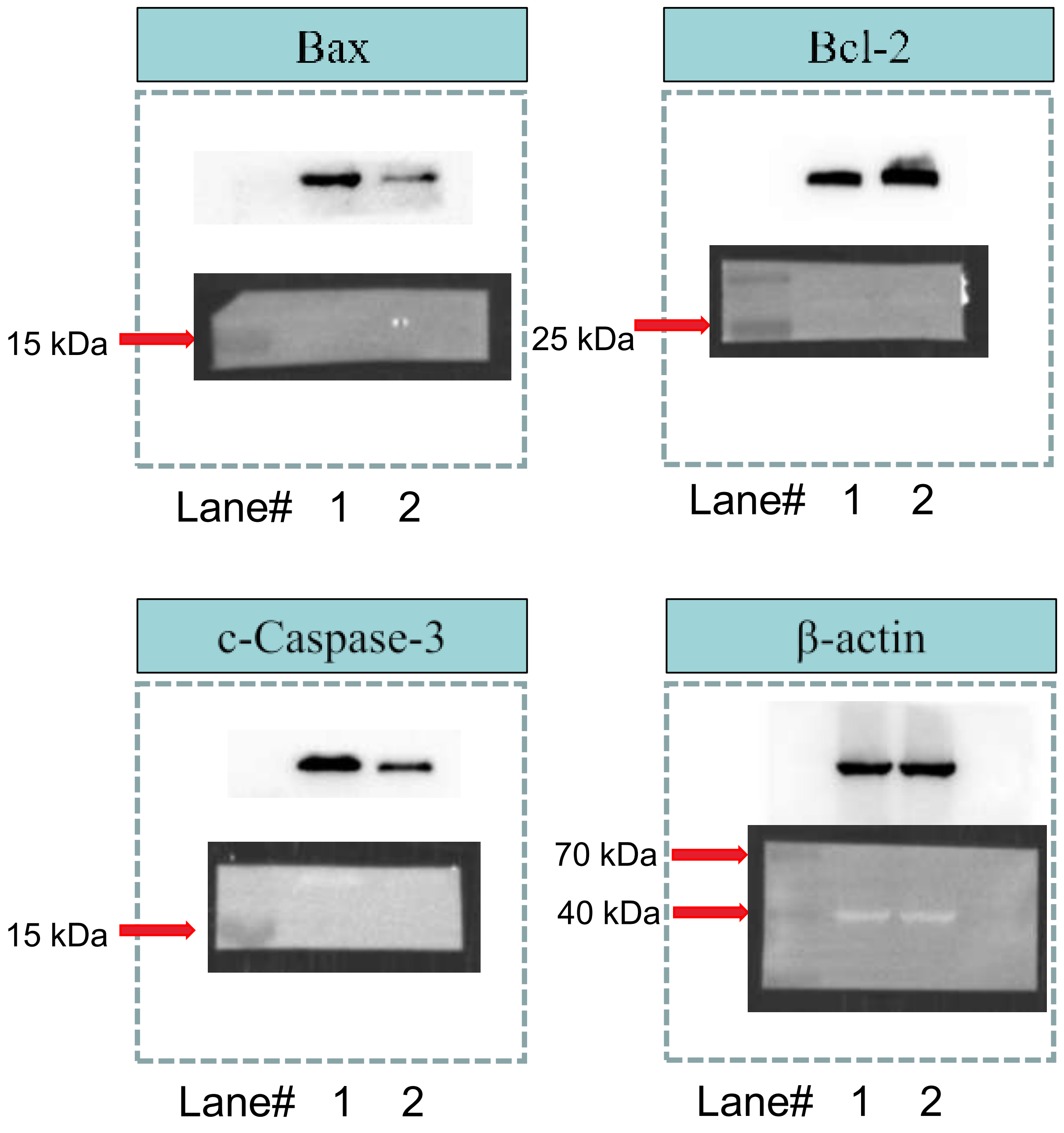

Figure 7I

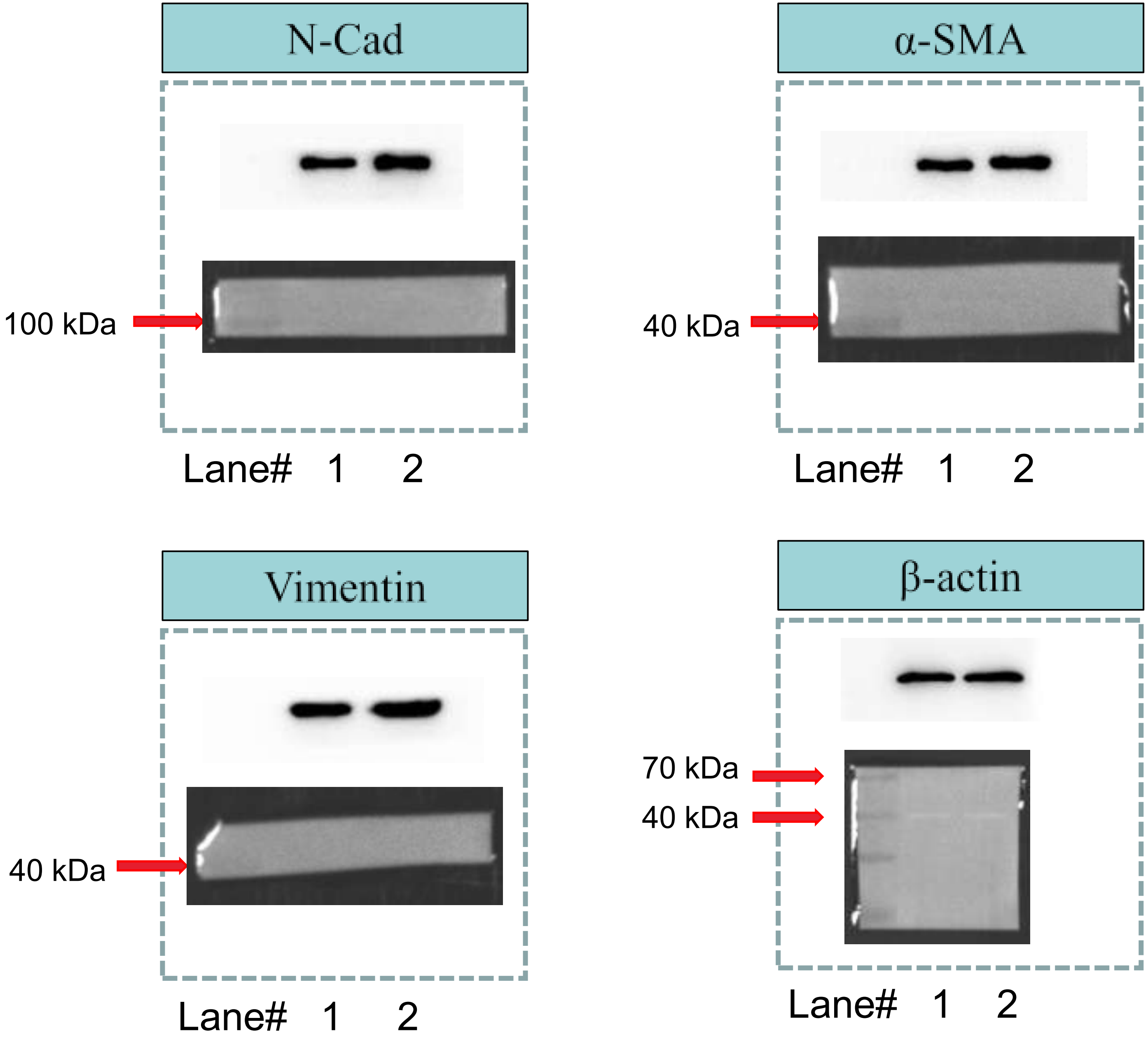
N-Cad
100 kDa
Lane# 1 2
α-SMA
40 kDa
Lane# 1 2
Vimentin
40 kDa
Lane# 1 2
β-actin
70 kDa
40 kDa
Lane# 1 2

Figure 8A

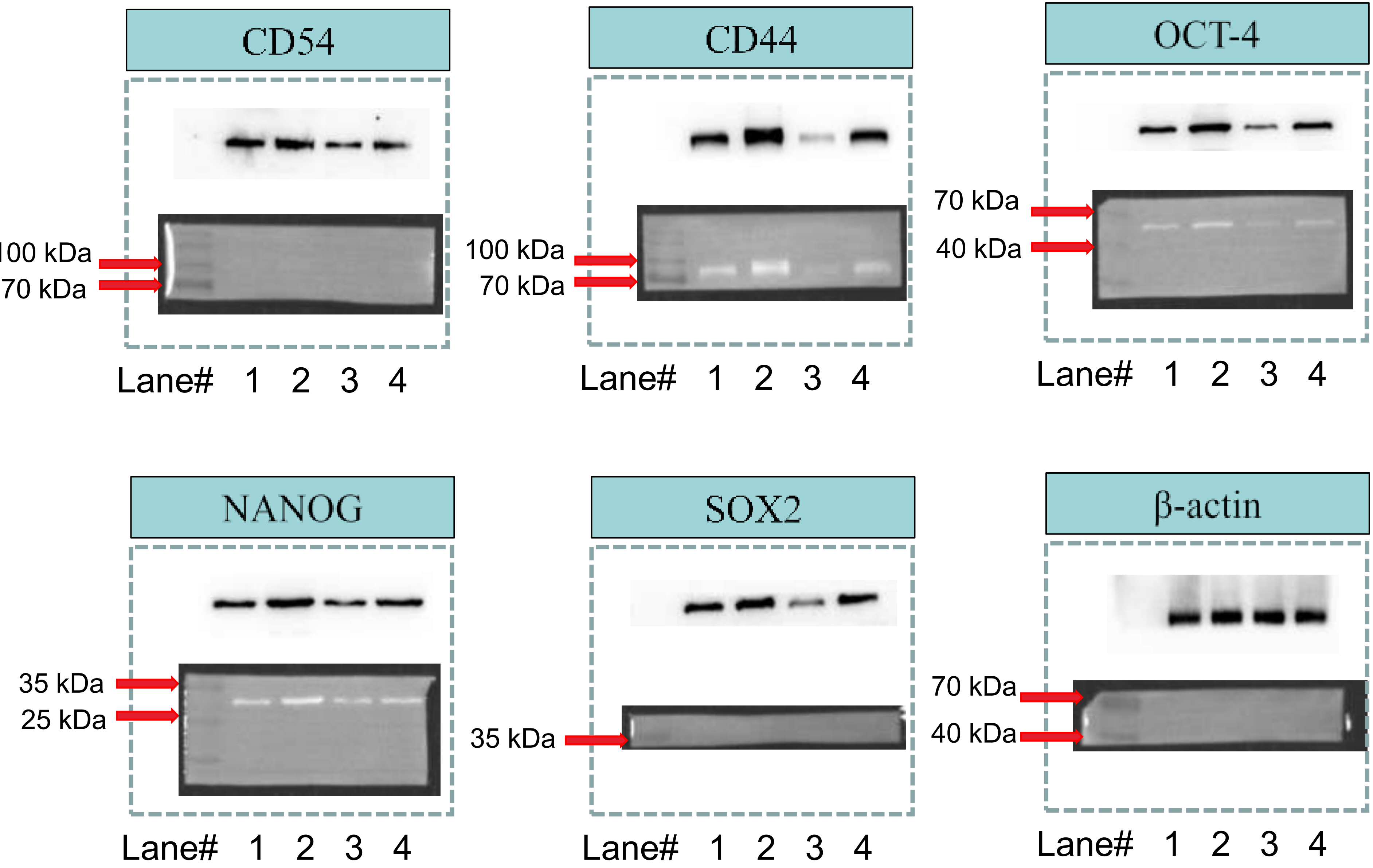
CD54
100 kDa
70 kDa
Lane# 1 2 3 4
CD44
100 kDa
70 kDa
Lane# 1 2 3 4
OCT-4
70 kDa
40 kDa
Lane# 1 2 3 4
NANOG
35 kDa
25 kDa
Lane# 1 2 3 4
SOX2
35 kDa
Lane# 1 2 3 4
β-actin
70 kDa
40 kDa
Lane# 1 2 3 4

Figure 8B

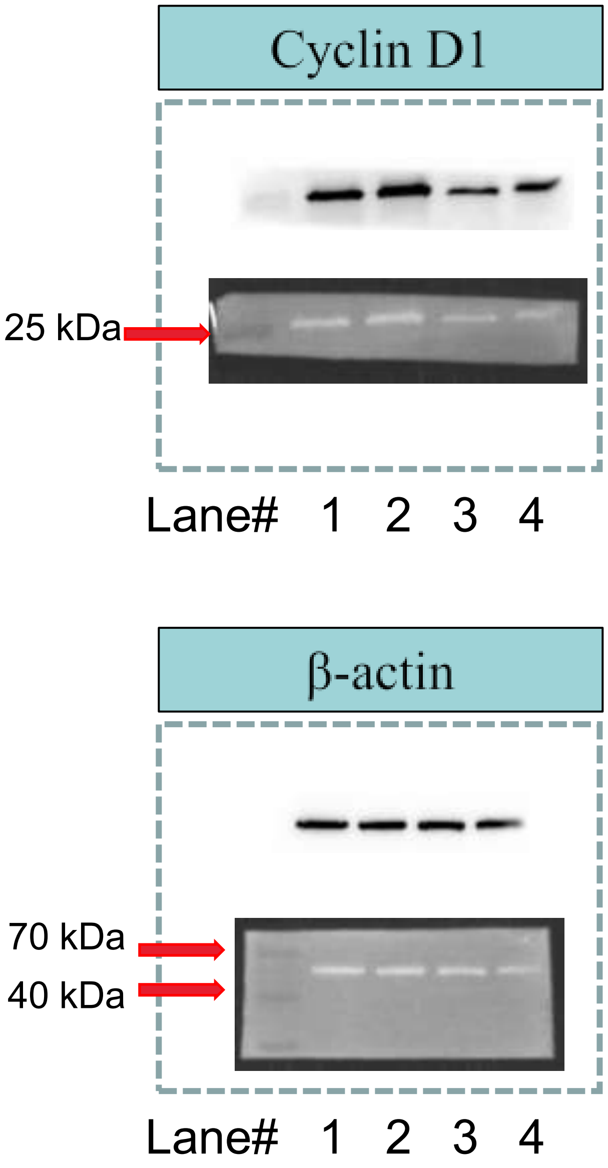

Figure 8C

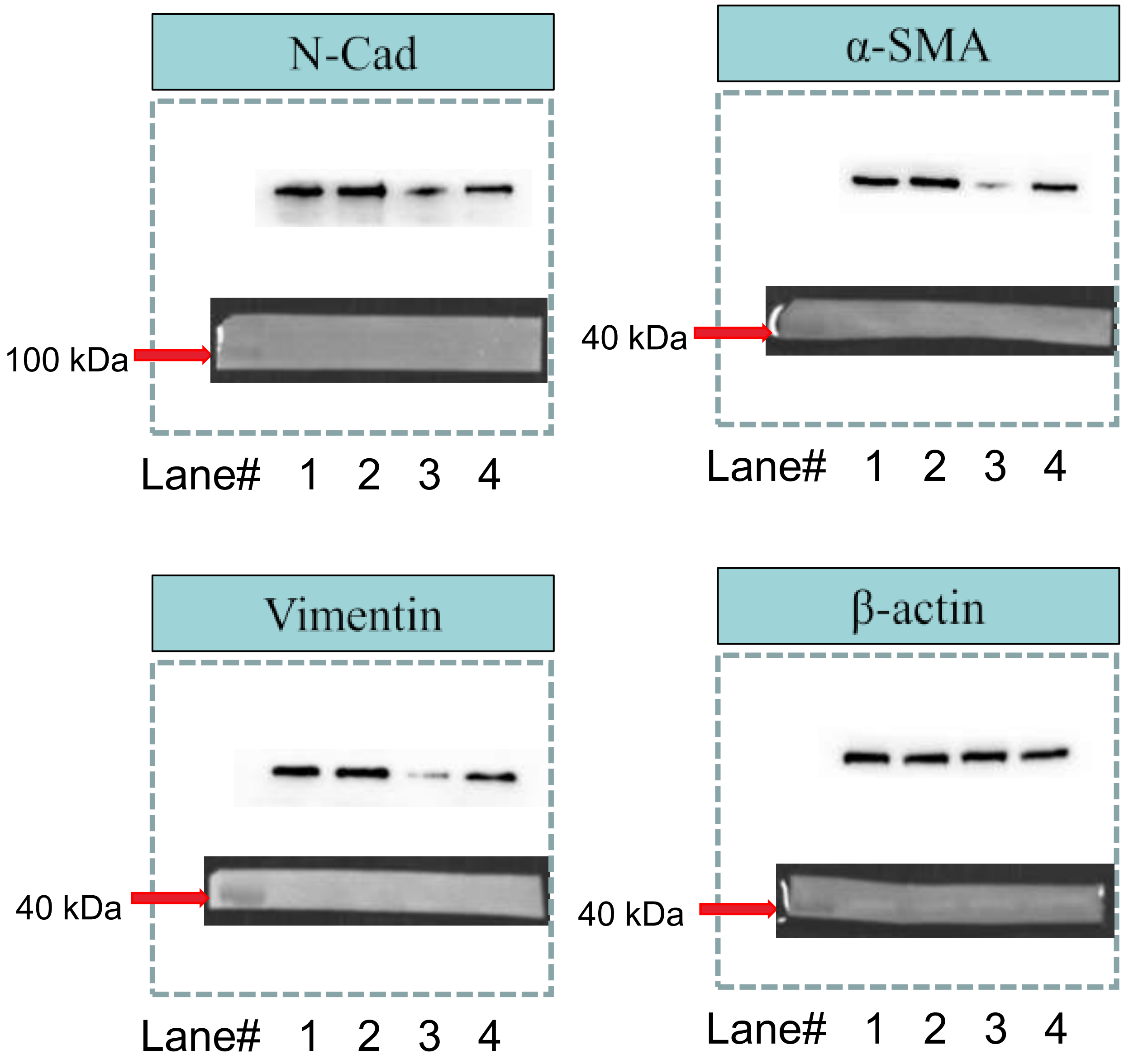

Figure S1B

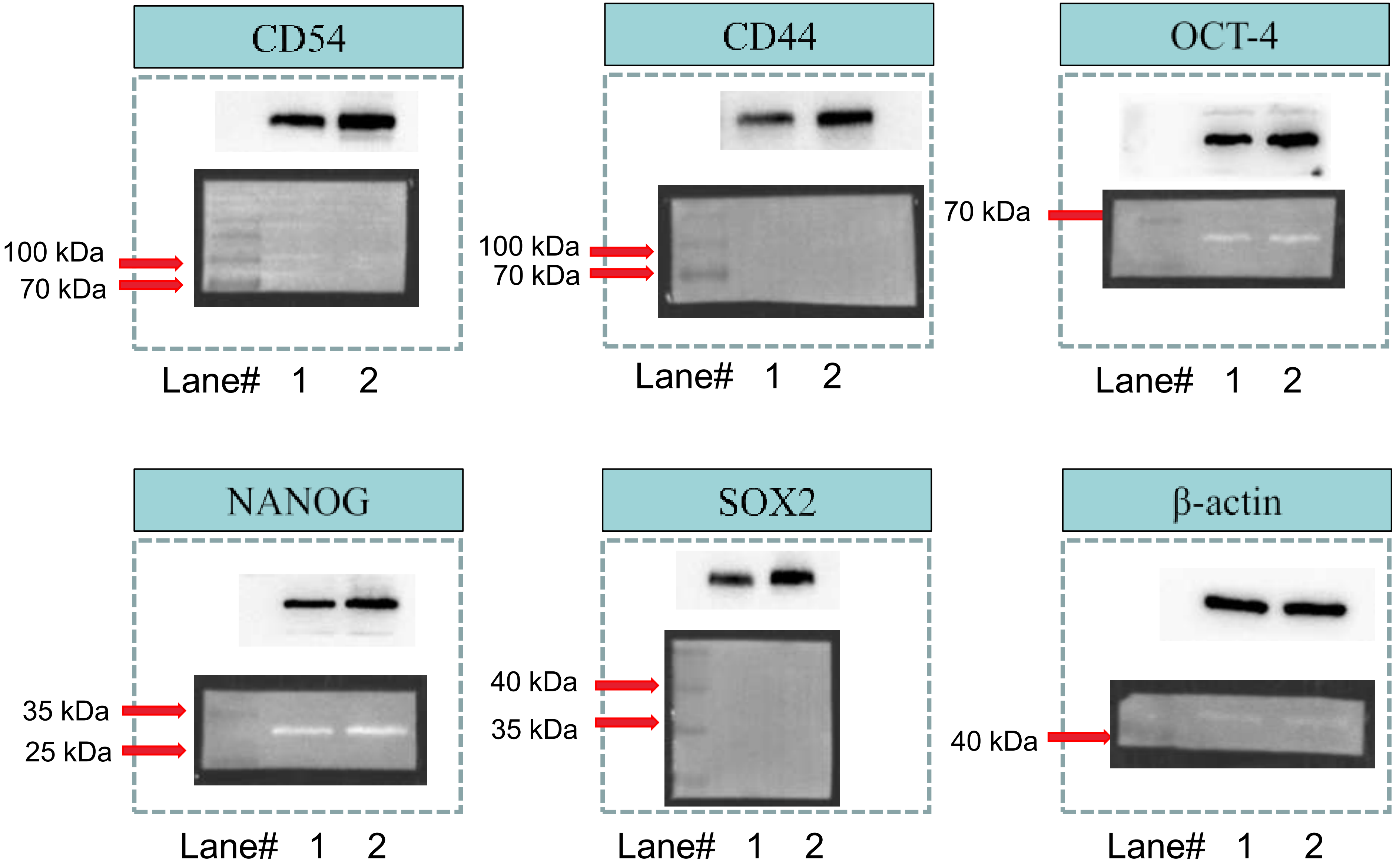
CD54
100 kDa
70 kDa
Lane# 1 2
CD44
100 kDa
70 kDa
Lane# 1 2
OCT-4
70 kDa
Lane# 1 2
NANOG
35 kDa
25 kDa
Lane# 1 2
SOX2
40 kDa
35 kDa
Lane# 1 2
β-actin
40 kDa
Lane# 1 2

Figure S1G

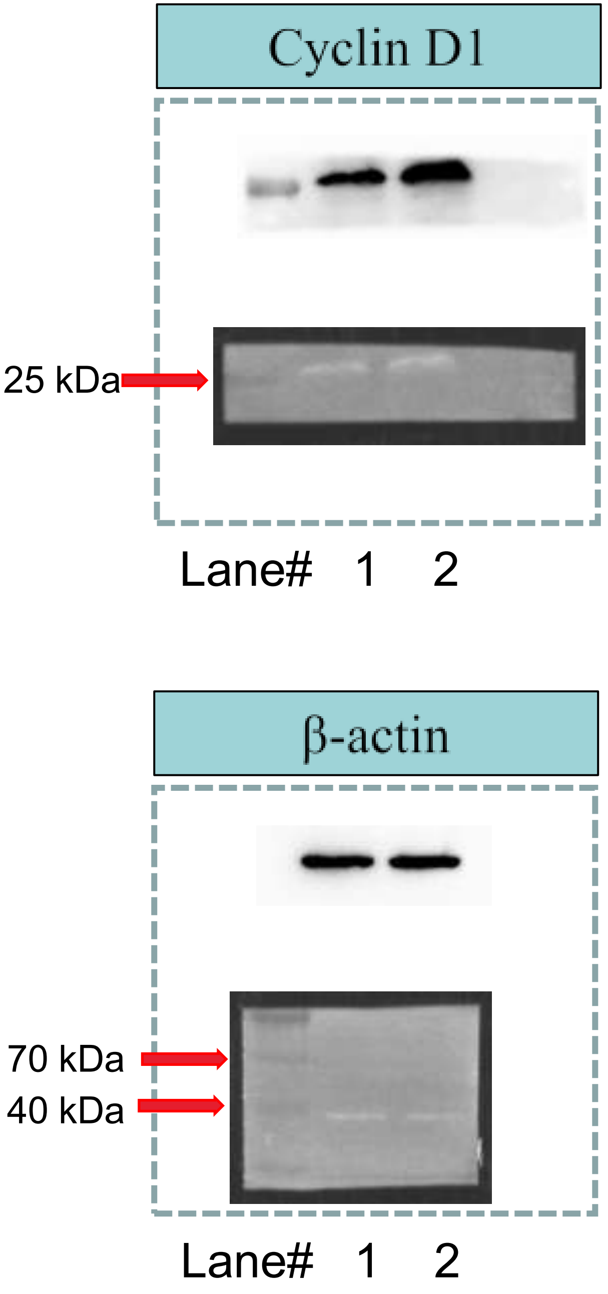

Figure S1I

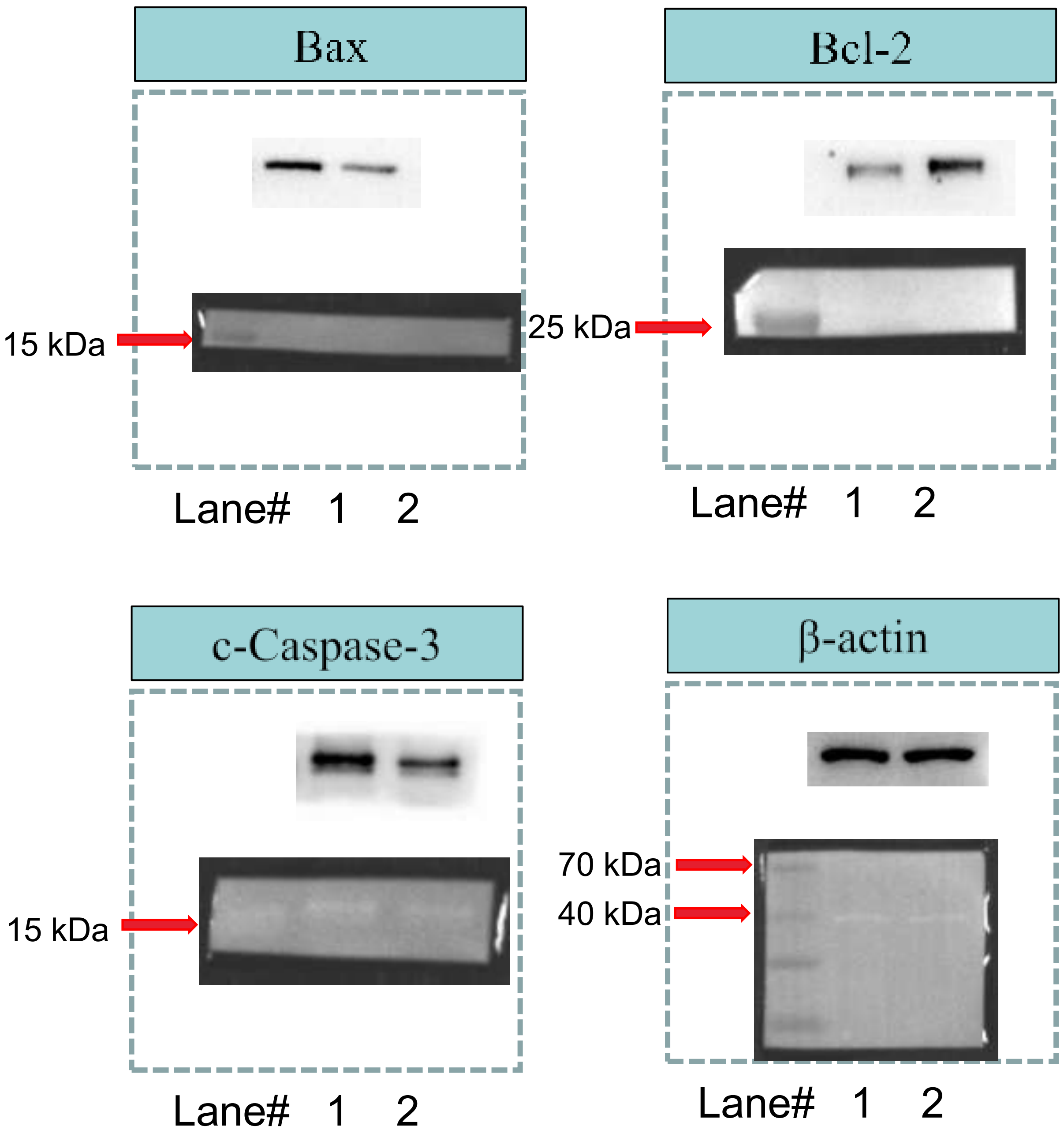

Figure S1J

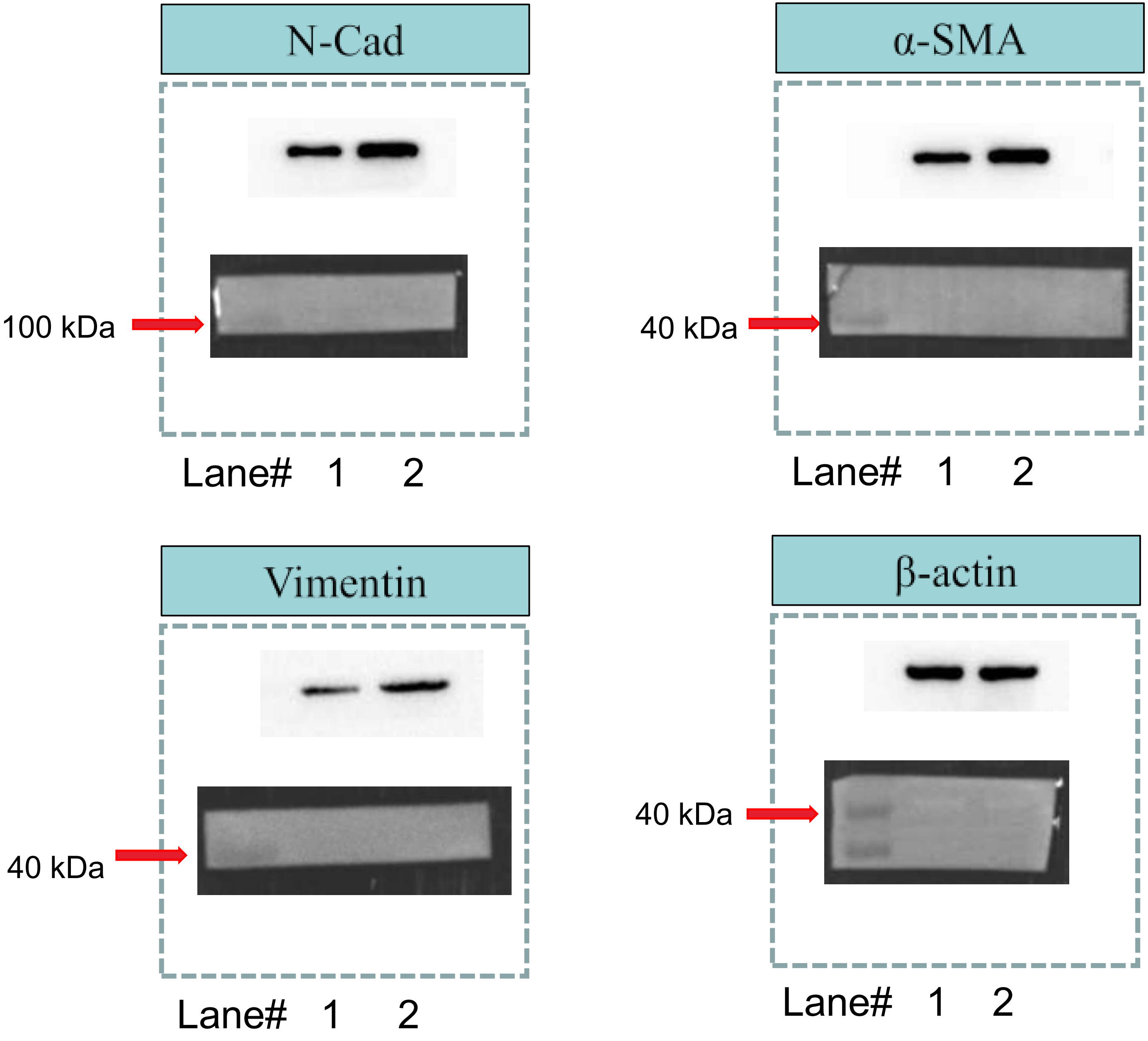
N-Cad
100 kDa
Lane# 1 2
α-SMA
40 kDa
Lane# 1 2
Vimentin
40 kDa
Lane# 1 2
β-actin
40 kDa
Lane# 1 2

Figure S3B

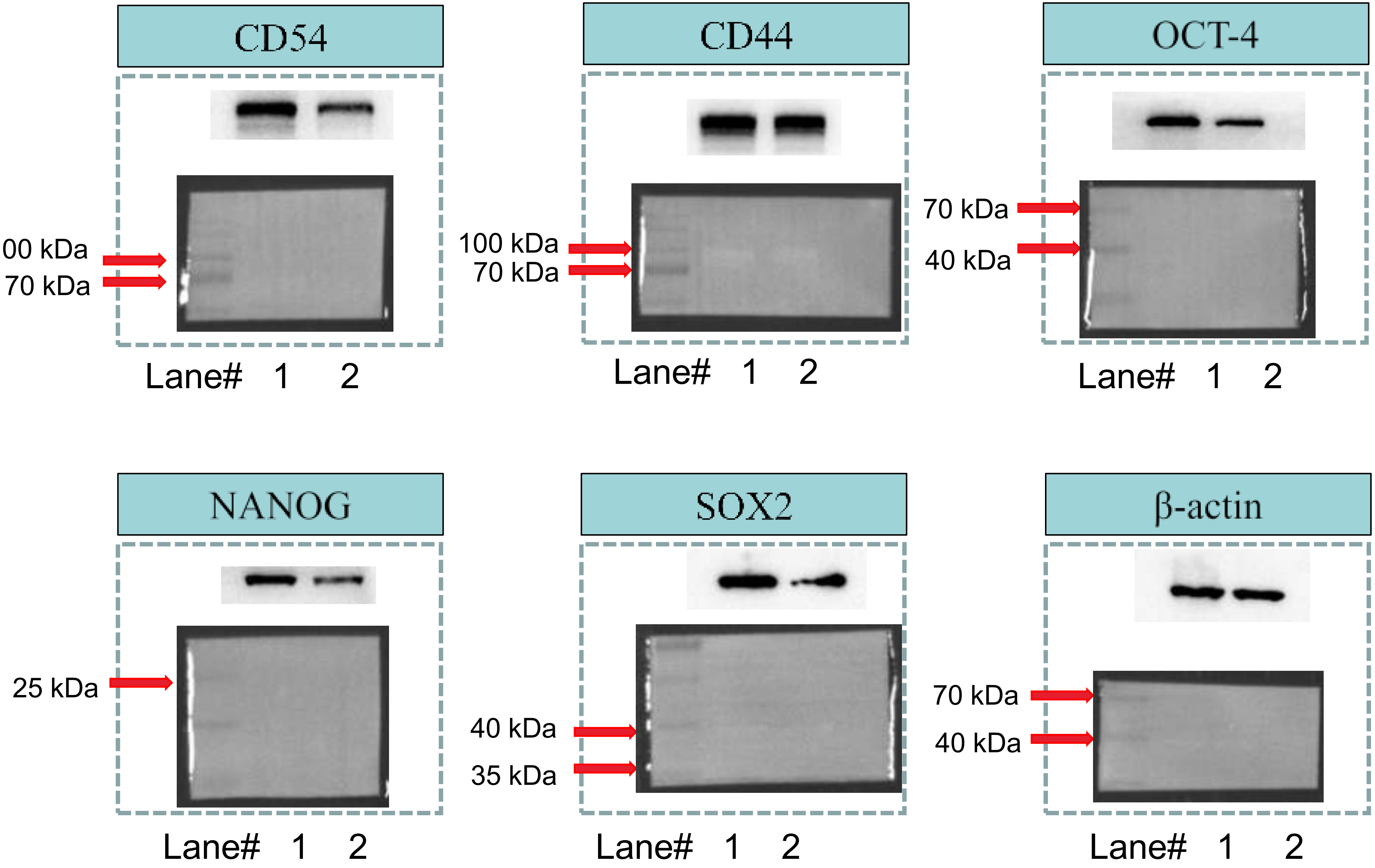
CD54
00 kDa
70 kDa
Lane# 1 2
CD44
100 kDa
70 kDa
Lane# 1 2
OCT-4
70 kDa
40 kDa
Lane# 1 2
NANOG
25 kDa
Lane# 1 2
SOX2
40 kDa
35 kDa
Lane# 1 2
β-actin
70 kDa
40 kDa
Lane# 1 2

Figure S3F

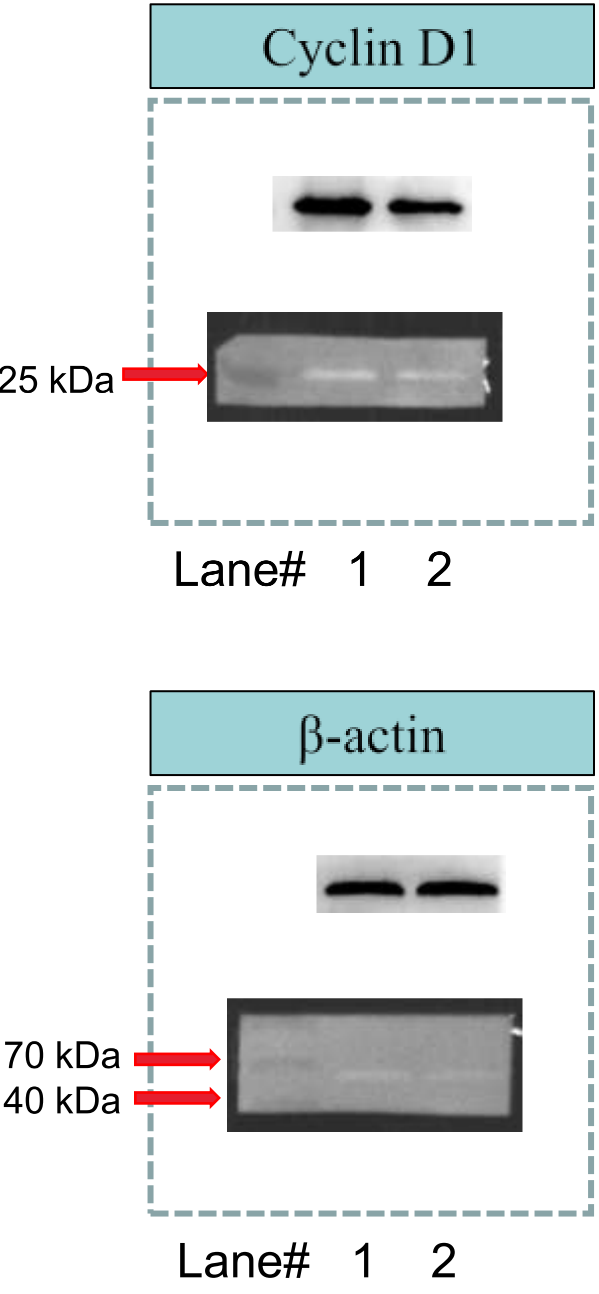

Figure S3H

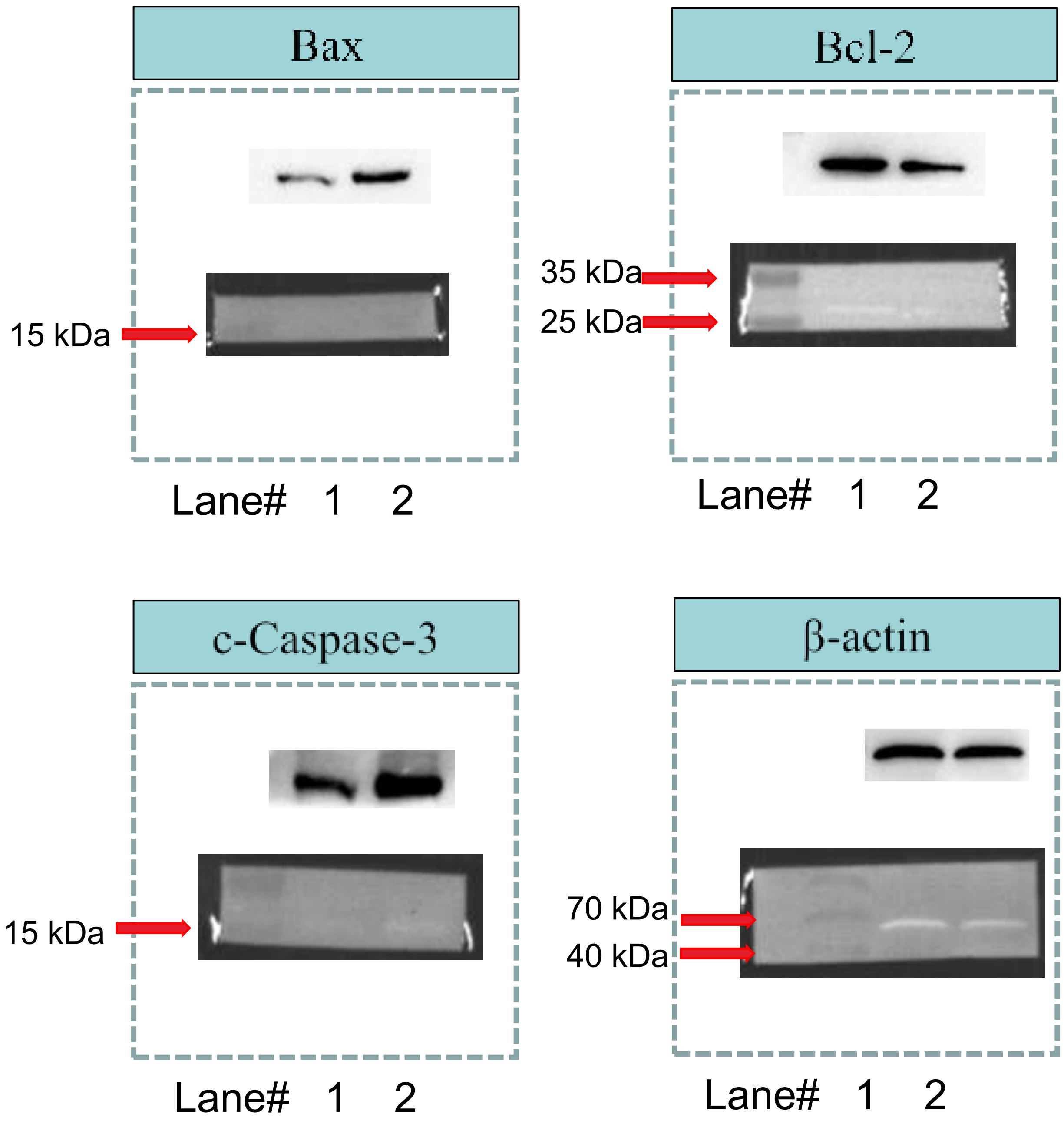

Figure S3I

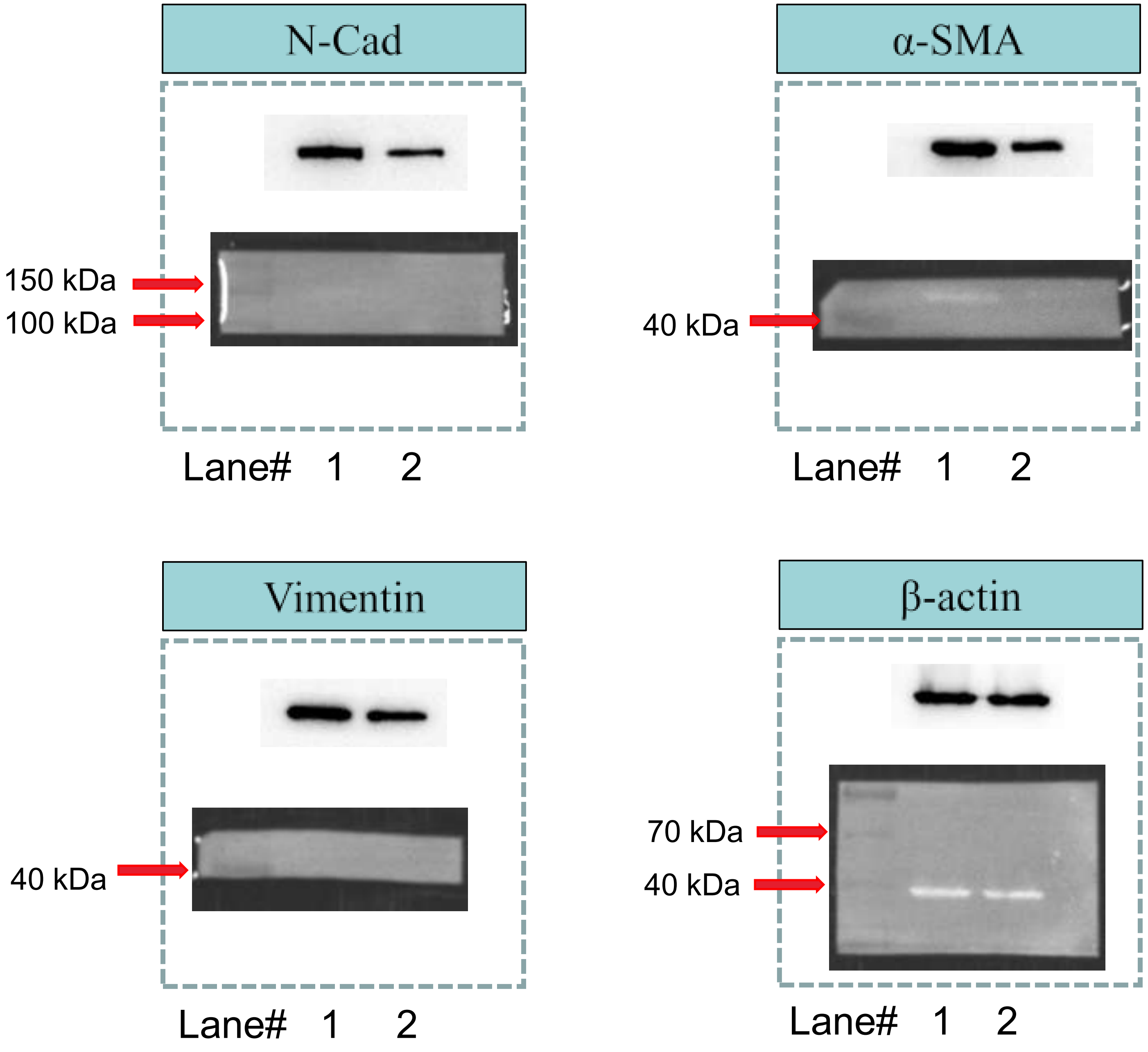
N-Cad
150 kDa
100 kDa
Lane# 1 2
α-SMA
40 kDa
Lane# 1 2
Vimentin
40 kDa
Lane# 1 2
β-actin
70 kDa
40 kDa
Lane# 1 2

Figure S4A

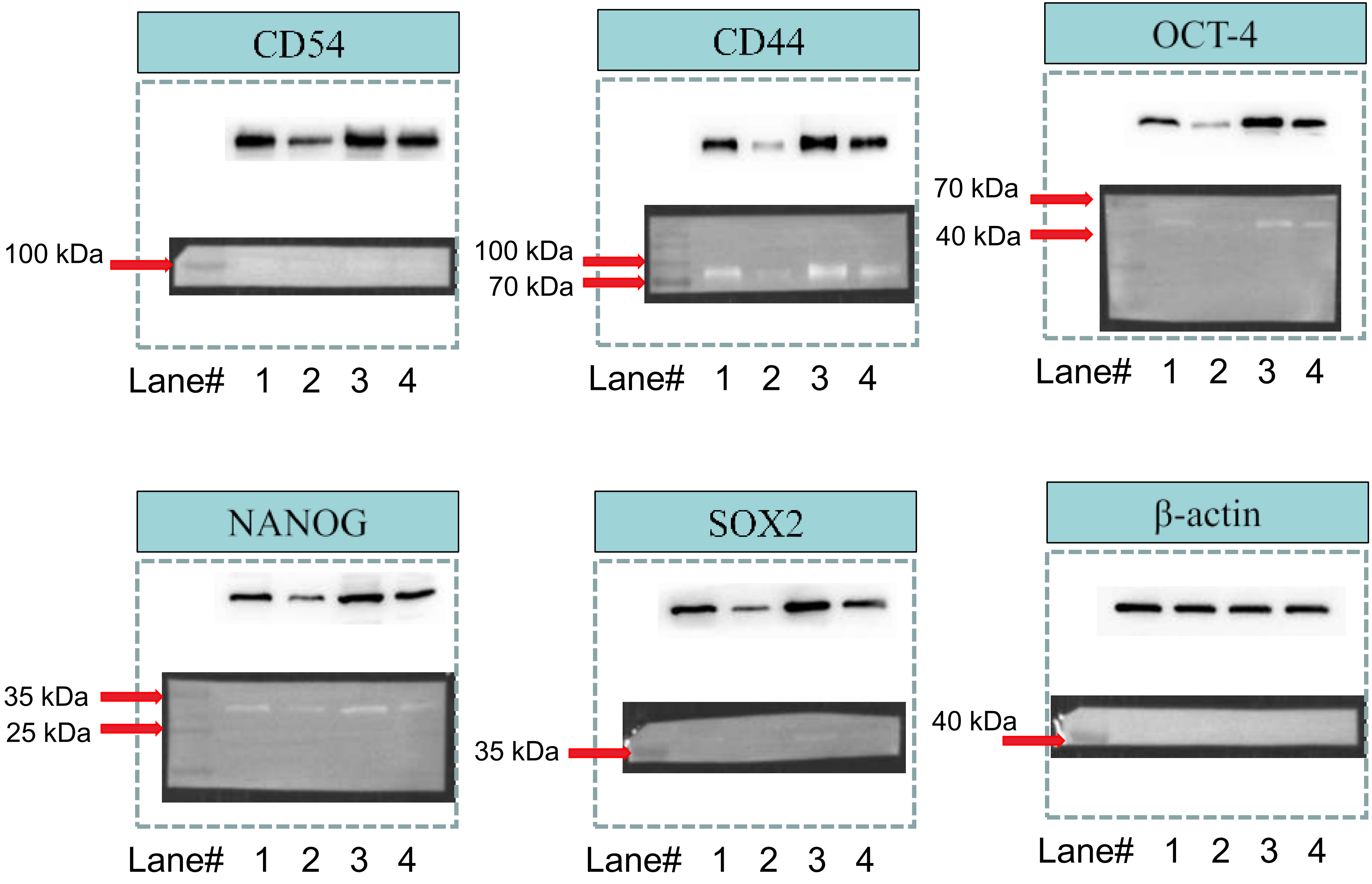

CD54
100 kDa
Lane# 1 2 3 4
CD44
100 kDa
70 kDa
Lane# 1 2 3 4
OCT-4
70 kDa
40 kDa
Lane# 1 2 3 4
NANOG
35 kDa
25 kDa
Lane# 1 2 3 4
SOX2
35 kDa
Lane# 1 2 3 4
β-actin
40 kDa
Lane# 1 2 3 4

Figure S4B

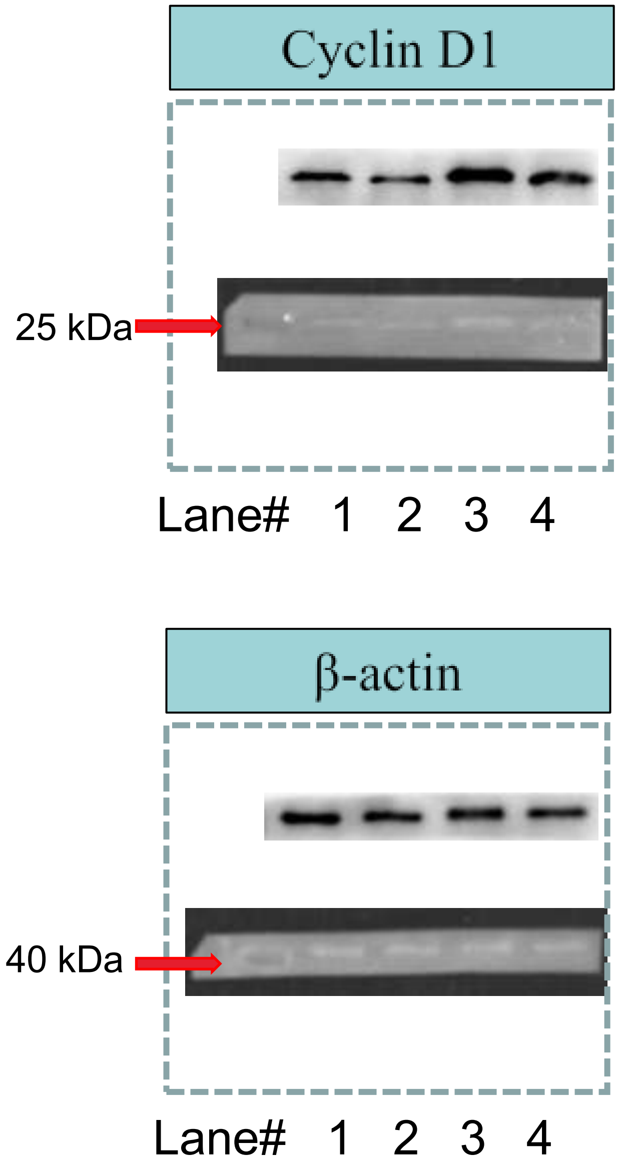

Figure S4C

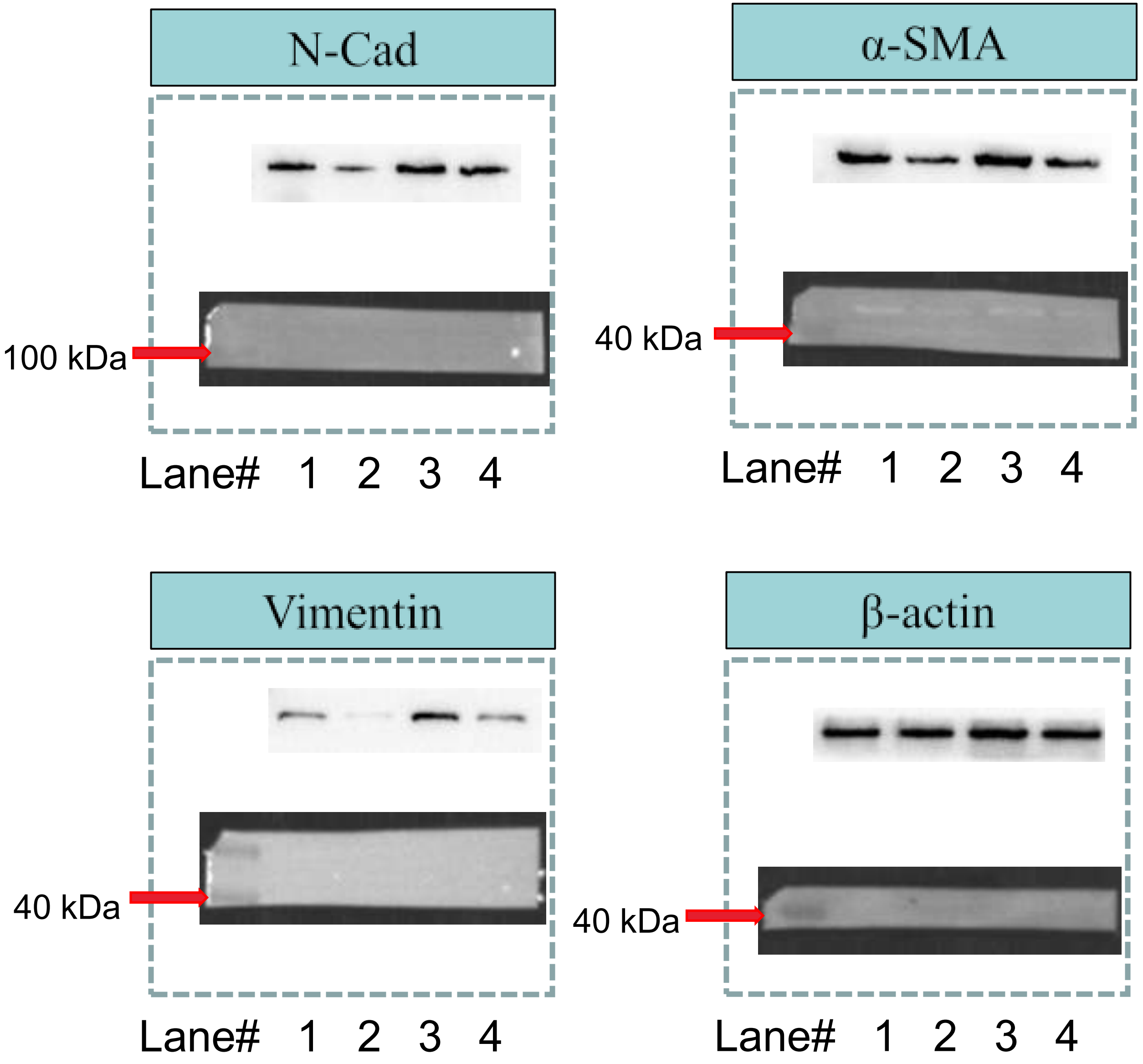

Supplement: Supplementary file 1 [file biomolecules-15-01227-s001.zip › biomolecules-3742892-Western blot.pdf]
