# Supplementary figures and images for: LncRNA TSPEAR-AS2 Maintains the Stemness of Gastric Cancer Stem Cells by Regulating the miR-15a-5p/CCND1 Axis
Source: Biomolecules. 2025 Aug 26;15(9):1227. doi: 10.3390/biom15091227 (PMC12467362; doi:10.3390/biom15091227)

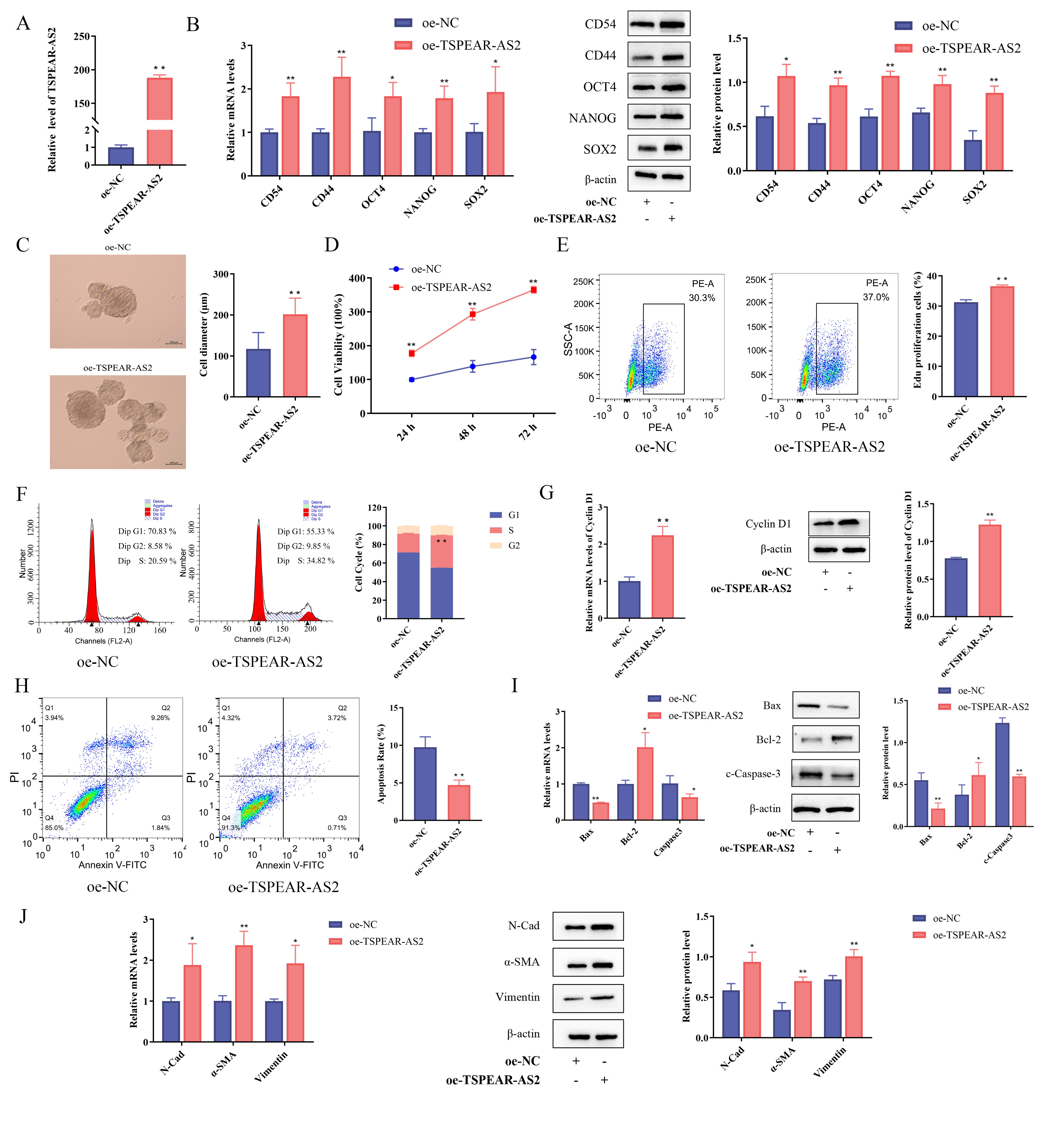

Supplement: Supplementary file 1 [file biomolecules-15-01227-s001.zip › Supplementary-Figure S1.jpg]

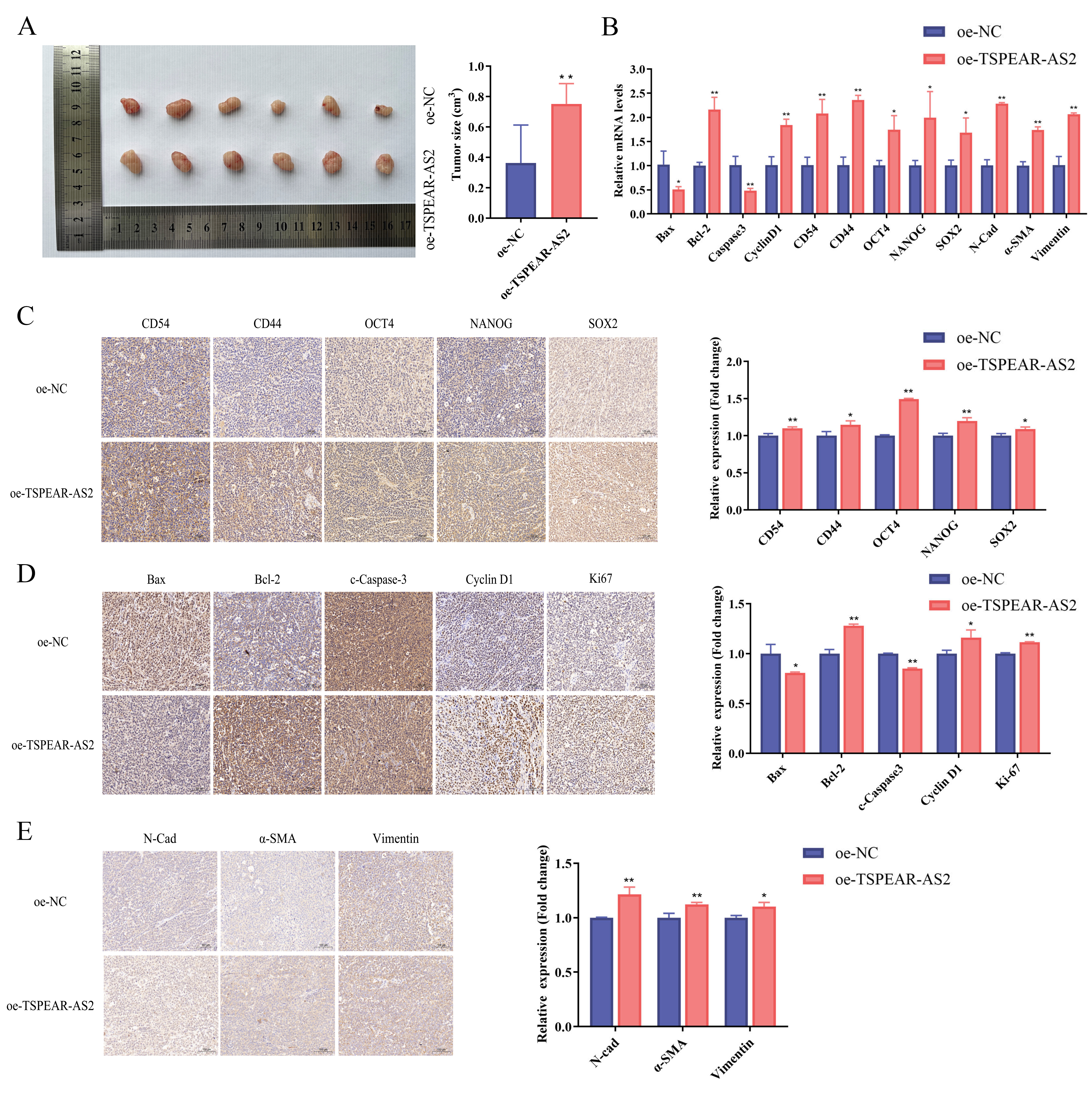

Supplement: Supplementary file 1 [file biomolecules-15-01227-s001.zip › Supplementary-Figure S2.jpg]

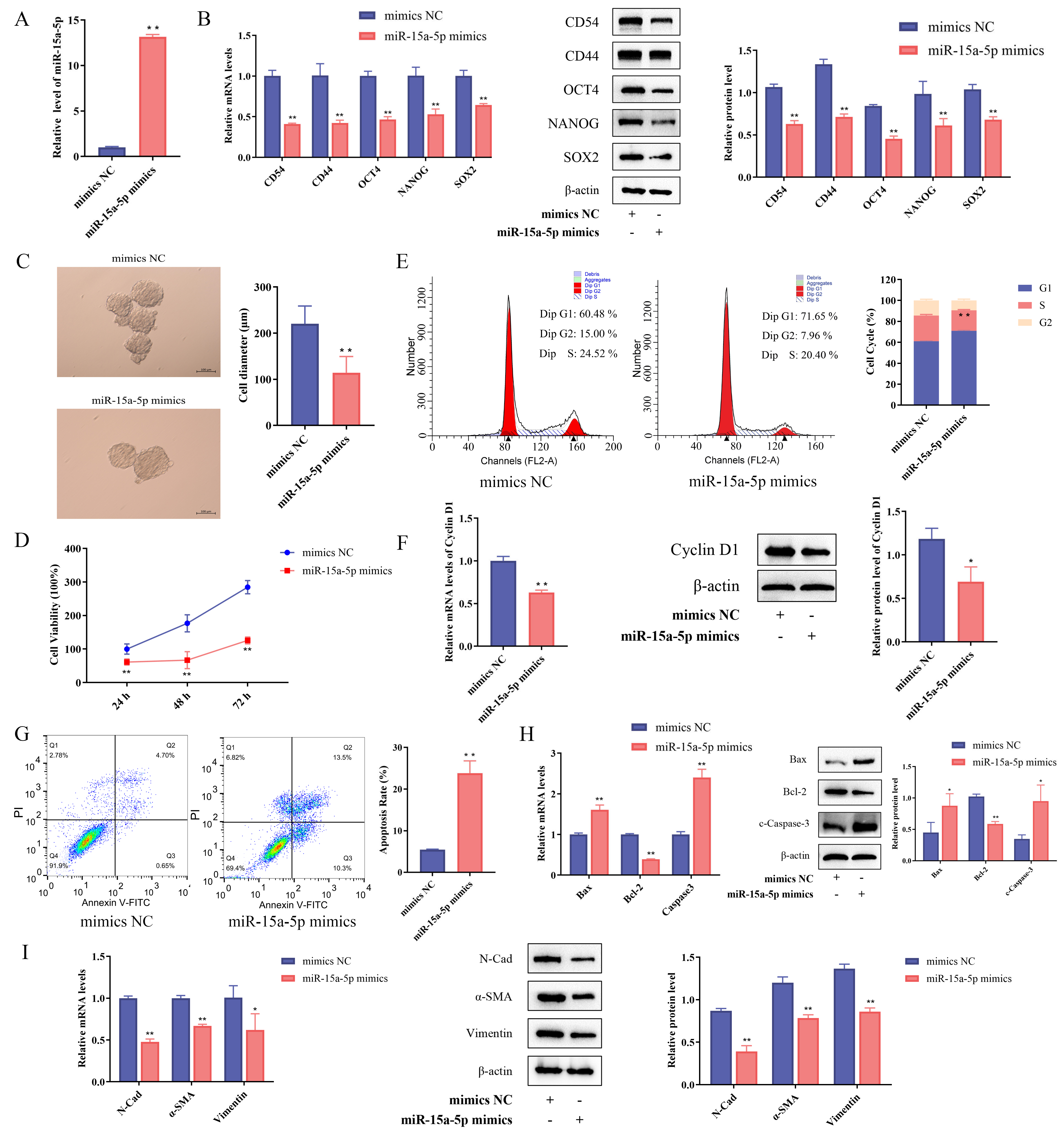

Supplement: Supplementary file 1 [file biomolecules-15-01227-s001.zip › Supplementary-Figure S3.jpg]

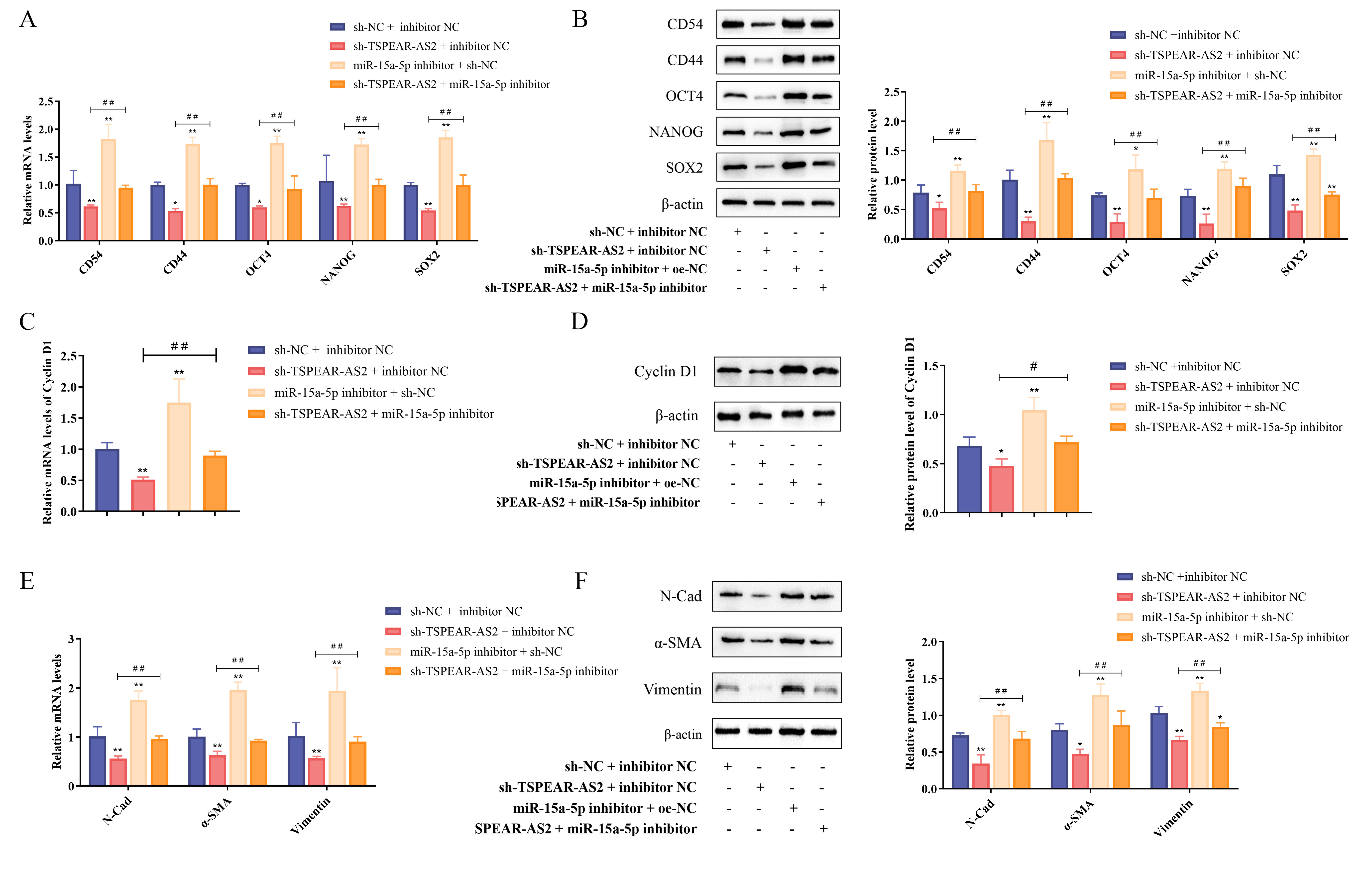

Supplement: Supplementary file 1 [file biomolecules-15-01227-s001.zip › Supplementary-Figure S4.jpg]
